# Generative Artificial Intelligence Optimization of Albumin Binders: Coumarin and Fatty Acid Derivatives

**DOI:** 10.1021/acs.jcim.6c00296

**Published:** 2026-06-02

**Authors:** Yihao Zhang, Qirui Deng, Xin Yang, Xinchen Yue, Huarui Zhang, Shijian Ding, Jinping Lei, Baoting Zhang, Sifan Yu, Ge Zhang

**Affiliations:** † Law Sau Fai Institute for Advancing Translational Medicine in Bone and Joint Diseases (TMBJ), School of Chinese Medicine, 26679 Hong Kong Baptist University, Kowloon, Hong Kong SAR 999077, China; ‡ School of Chinese Medicine, Faculty of Medicine, 26451 The Chinese University of Hong Kong, Kowloon, Hong Kong SAR 999077, China; § School of Pharmaceutical Sciences, 26469 Sun Yat-Sen University, Guangzhou 510006, China; ∥ Shenzhen Institute for Research and Continuing Education (IRACE), Hong Kong Baptist University, Shenzhen 518000, China

## Abstract

Previously, we reported a dual combination based on 4-hydroxycoumarin and dodecanedioic acid that could synergistically bind to human serum albumin (HSA). However, optimizing this combination remains challenging and could often be guided by empirical selection and extensive experimental screening, which may limit the chemical diversity and suboptimal affinity. In this study, we established a systematic artificial intelligence framework that integrates computational optimization with wet-lab synthesis and experimental validation, enabling improvement of the dual combination while preserving the core chemotypes. We first trained a machine learning classifier on curated HSA binding data and used it as an external scoring function to guide reinforcement learning-driven scaffold decoration with LibINVENT, enabling goal-directed generation of coumarin derivatives and fatty acid derivatives. Candidate molecules were prioritized through multiparameter filtering and diversity-aware selection, followed by synthesis and experimental validation using surface plasmon resonance. The optimized representatives show nanomolar HSA binding and enhanced affinity compared to the original ligands. Molecular docking and molecular dynamics simulations further provide a mechanistic rationale for the affinity improvements by revealing additional stabilizing interactions and more favorable binding energetics at the corresponding HSA sites. Besides, the optimized coumarin derivative (CD1) is a warfarin-derived coumarin analogue, yet it did not show detectable anticoagulant activity in an acute clotting time assay, whereas warfarin did. Overall, this work demonstrates a practical AI-guided route to expand chemical diversity and improve affinity for a synergistic HSA binding combination.

## Graphical abstract

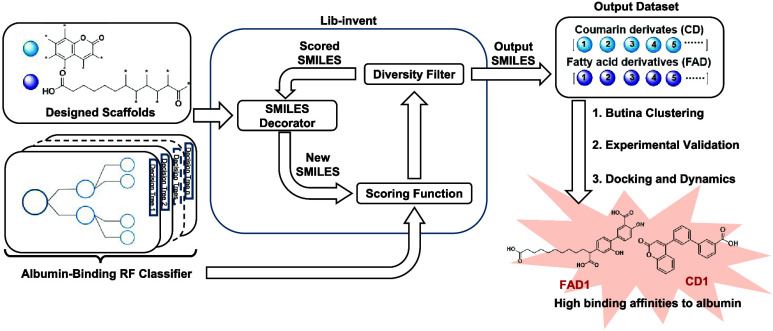

## 1 Introduction

Human serum albumin (HSA) is the most abundant plasma protein with a molecular weight of approximately 66 kDa and an exceptionally long circulation half-life.^1^ Owing to its high *in vivo* stability and multiple ligand binding regions, HSA has been widely exploited as an endogenous carrier to prolong systemic exposure of therapeutics.^2^ In practice, many drugs and drug conjugates extend their circulation time by forming albumin-drug complexes through covalent conjugation or noncovalent binding.^3^ Previously, we designed a dual combination comprising a warfarin derivative, 4-hydroxycoumarin (HC), and a fatty acid derivative, dodecanedioic acid (DA), which could synergistically bind to HAS.^4^ Importantly, conjugating this dual combination to a nucleic acid aptamer could extend the *in vivo* half-life of the conjugated aptamer, which helps address the druggability challenge of aptamers with short half-life.^5^ These findings motivate the systematic optimization of the dual combination to enhance HSA binding while preserving the core chemotypes.

However, optimizing these combinations remains challenging. Conventional optimization approaches are often guided by empirical selection together with extensive experimental screening, which can be time-consuming and labor-intensive.^6^ More importantly, empirical workflows tend to explore only a narrow fraction of the chemical space around the starting ligands, which may limit chemical diversity and constrain the achievable affinity gains.^7^ These limitations create a need for a systematic strategy that can efficiently expand chemical diversity while prioritizing candidates with improved levels of albumin binding.

Artificial intelligence (AI) offers a systematic route to expand chemical diversity and prioritize higher-quality candidates.^8^ While traditional virtual screening is largely restricted to existing molecular libraries, deep generative models can systematically expand analog space around known chemotypes.^9^ In our case, this enables the exploration of a much broader chemical neighborhood surrounding HC and DA scaffolds to identify derivatives with improved properties while retaining the core structures. Specifically, integrating robust predictive algorithms such as random forest with scaffold-focused generative frameworks such as LibINVENT establishes a practical closed-loop workflow.^10^ In this setup, the generative model proposes chemically feasible scaffold decorations and analog candidates, while the predictive model acts as a surrogate scorer that guides generation toward a higher albumin binding likelihood. This coupled strategy reduces reliance on exhaustive trial and error screening and provides a rational, data-driven pathway to optimize the dual combination for HSA binding.

In this study, we established a systematic AI framework that integrates computational optimization with wet-lab synthesis and experimental validation, enabling improvement of the dual combination while preserving the core chemotypes. We trained a machine learning classifier on curated HSA binding data and used it as an external scoring function to guide reinforcement learning-driven scaffold decoration with LibINVENT, enabling goal-directed generation of coumarin derivatives (CDs) and fatty acid derivatives (FADs) (Figure 1). Candidate molecules were prioritized using a multistage selection pipeline, including physicochemical filtering, predicted binding probability thresholding, and diversity-aware clustering to reduce redundancy and preserve chemical coverage. From the top-scoring and most populated clusters, we selected representative centroid molecules with favorable synthetic feasibility and designated them as CD1 and FAD1 for wet-lab synthesis and SPR validation. We further applied molecular docking and molecular dynamics simulations to provide a mechanistic rationale for affinity improvements by analyzing stabilizing interactions and binding energetics at the corresponding HSA sites. Besides, because warfarin-related chemotypes can raise concerns about anticoagulant liability, we performed an *in vivo* acute clotting time assay as a preliminary safety screen and found that the optimized coumarin derivative CD1, although warfarin-derived, did not show detectable anticoagulant activity under the tested conditions, whereas warfarin did. Overall, this work demonstrates a practical AI-guided route to expand chemical diversity and improve affinity for a synergistic HSA binding combination.

**Figure 1:**
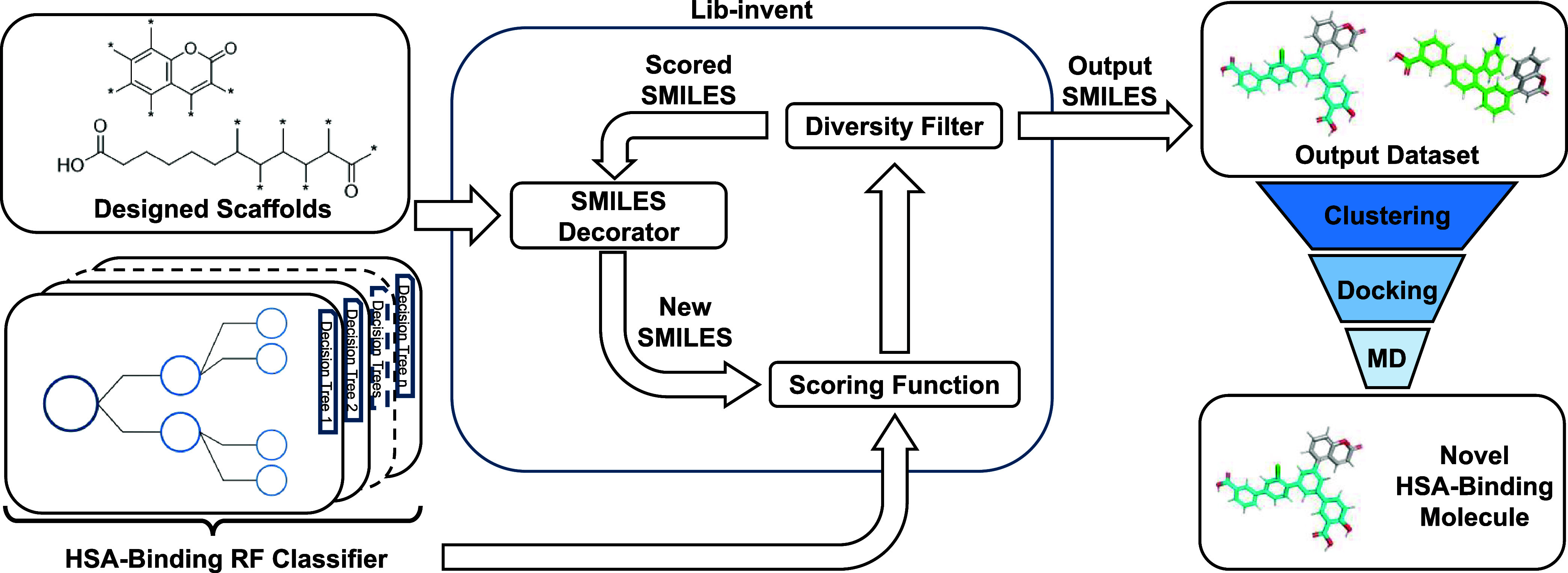
Schematic representation of the overall workflow.

## 2 Experimental Methods

### 2.1 Data Set Construction and Preprocessing

To construct a robust training set for the HSA-binding predictor, we curated high-affinity ligand data from public repositories, including BindingDB^11^ and PDBbind+.^12^ Positive samples were defined as molecules exhibiting a dissociation constant (KD) or inhibition constant (*K*_i_) of less than 1 mM against human serum albumin. The initial collection was filtered to remove macromolecules, retaining only small molecules with a molecular weight (MW) of less than 1000 Da. This yielded 185 unique high-affinity binders. To balance the data set and enable discriminative learning, 555 putative nonbinders (decoys) were randomly sampled from the ZINC15 database^13^ using stratified sampling to maintain a positive-to-negative ratio of 1:3. Molecular structures were standardized and canonicalized by using the RDKit library prior to feature extraction.

### 2.2 Molecular Featurization

Structural information was encoded into numerical vectors using extended connectivity fingerprints (ECFPs). Specifically, Morgan fingerprints were generated by using RDKit with a radius of 3 (equivalent to ECFP6) and mapped to a fixed-length bit vector of 2048 bits. This representation captures circular substructural features up to a diameter of 6 bonds, ensuring sufficient resolution for ligand similarity comparisons (Figure 2).

**Figure 2:**
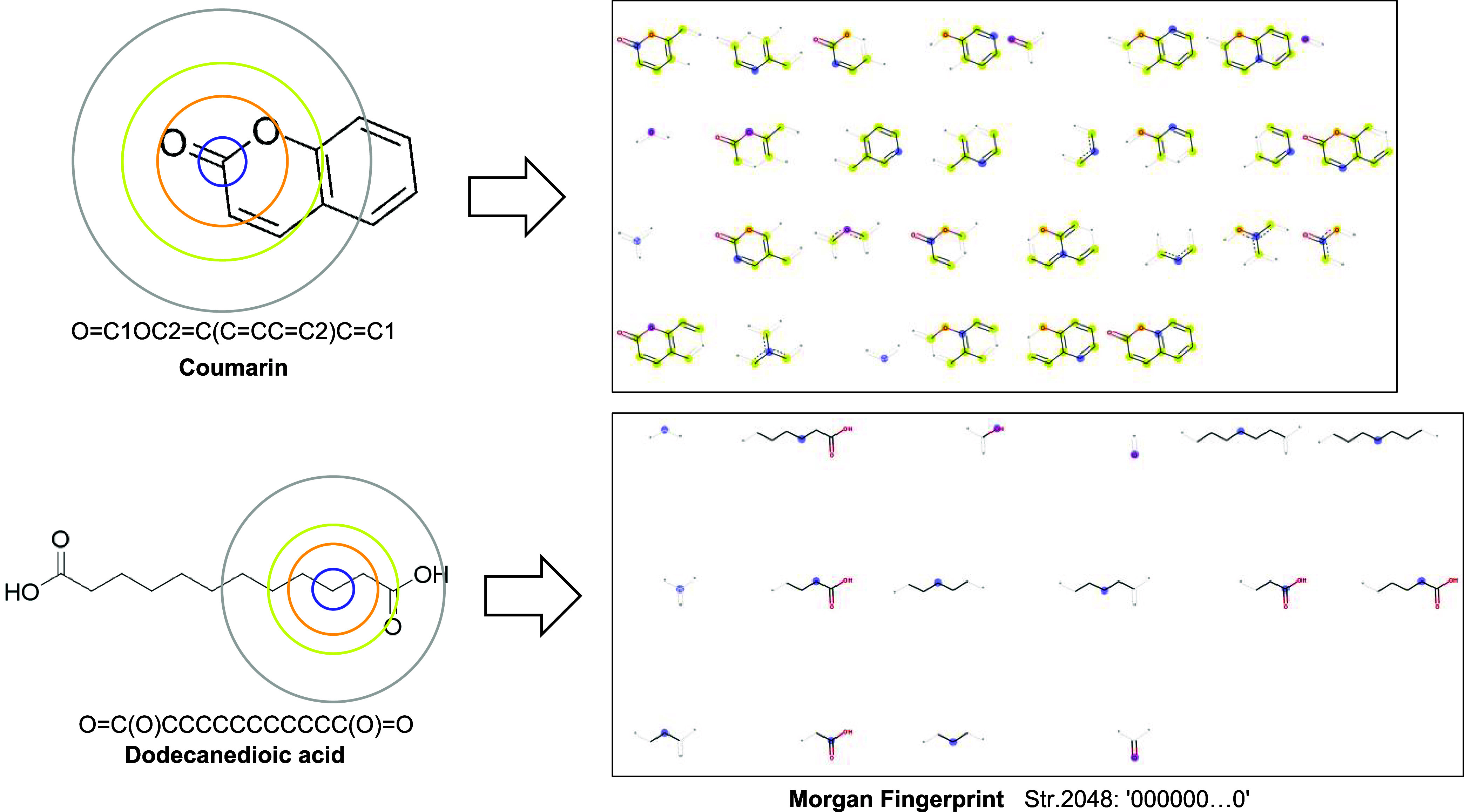
Illustration of Morgan fingerprints. Visualization of Morgan fingerprints using coumarin and dodecanedioic acid as examples.

### 2.3 Machine Learning Framework: Random Forest Classifier

Using the ECFP6 fingerprints (Morgan fingerprints, radius = 3, 2048 bits) generated in Section 2.2 as input features, we built a predictive model using the Random Forest (RF) algorithm implemented in scikit-learn.^14^ The data set was randomly partitioned into a training set (80%) and an independent test set (20%). Hyperparameter optimization was conducted using the Optuna framework to tune critical parameters, including the number of estimators (n_estimators = 241), maximum tree depth (max_depth = 29), and maximum features (max_features = 15).^15^ The final model was trained by using a 5-fold cross-validation strategy to mitigate overfitting and ensure generalizability. Performance metrics, including accuracy, precision, recall, F1-score, and area under the ROC curve (AUC-ROC), were calculated to evaluate model robustness.

### 2.4 Generative Deep Learning with Libinvent

We employed the Libinvent framework, a recurrent neural network (RNN)-based generative model designed for scaffold decoration.^10^ The previously trained RF classifier was integrated as an external scoring function to guide the reinforcement learning (RL) process. Two distinct scaffolds, coumarin and a fatty acid backbone, were defined as inputs for the SMILES decorator. During the RL phase, the model was incentivized to generate valid SMILES strings that maximized the predicted probability of HSA binding. The generation process was monitored until the average reward score converged.

### 2.5 Virtual Screening and Clustering Analysis

The generated library underwent a multistage virtual screening pipeline. First, compounds were filtered based on physicochemical properties (MW < 500 Da) and a high predicted binding score (>0.8). To ensure structural diversity among the leads, the surviving candidates were clustered using the Butina algorithm^16^ based on Tanimoto similarity indices calculated from Morgan fingerprints. The data set structure was visualized using t-distributed stochastic neighbor embedding (t-SNE).^17^ The centroid molecule from the top-ranking cluster of each scaffold series was selected as the lead candidate for the synthesis.

### 2.6 Chemical Synthesis

CD1 and FAD1 were synthesized as outlined in Figures S1 and S3, respectively. Their characterization results are provided in Figures S2 and S4, respectively.

#### 2.6.1 Preparation of 3-[3-(2-Oxochromen-4-yl) phenyl]­benzoic Acid (CD1)

##### 2.6.1.1 **Step 1:** Preparation of 3-[3-(4, 4, 5, 5-Tetramethyl-1,3,2-dioxaborolan-2-yl)­phenyl]­benzoic Acid

A mixture of 3-(3-bromophenyl)­benzoic acid (100 mg, 0.360 mmol), B_2_Pin_2_ (137.46 mg, 0.541 mmol), Pd_2_(dba)_3_ (33.04 mg, 0.036 mmol), tricyclohexylphosphane (30.36 mg, 0.108 mmol), and KOAc (106.25 mg, 1.08 mmol) in dioxane (2 mL) was stirred at 100 °C for 12 h under N_2_ atmosphere. LCMS showed 58% of peak with desired mass. The reaction liquid is used directly for the next step.

##### 2.6.1.2 **Step 2:** Preparation of 3-[3-(2-Oxochromen-4-yl) phenyl]­benzoic Acid

A mixture of 4-chlorochromen-2-one (50 mg, 0.276 mmol), 3-[3-(4,4,5,5-tetramethyl-1,3,2-dioxaborolan-2-yl)­phenyl]­benzoic acid (98.73 mg, 0.304 mmol), Na_2_CO_3_ (88.04 mg, 0.830 mmol), Pd­(PPh_3_)_4_ (31.99 mg, 0.027 mmol) in dioxane (3 mL), and H_2_O (0.6 mL) was degassed and purged with N_2_ three times, and then the mixture was stirred at 90 °C for 2 h under N_2_ atmosphere. LCMS showed 37% of peak with desired mass. The reaction mixture was poured into water (5 mL), extracted with ethyl acetate (5 mL × 2), the aqueous phase was adjusted to pH = 6 with HCl aqueous solution, extracted with ethyl acetate (5 mL × 2), washed with brine (5 mL), dried with Na_2_SO_4_, filtered, and concentrated under reduced pressure to give a crude product. The crude product was purified by prep-HPLC (column: Phenomenex Luna C18 150 mm × 25 mm × 10μm; mobile phase: [H_2_O­(0.225% FA)-ACN]; gradient:35%–65% B over 15.0 min) to give 3-[3-(2-oxochromen-4-yl) phenyl] benzoic acid (73.43 mg, 76.69% yield) as a white solid. ^1^H NMR (400 MHz, DMSO-*d*_6_) δ13.49–12.81 (m, 1H), 8.27 (s, 1H), 8.03 (d, *J* = 7.6 Hz, 1H), 7.97 (d, *J* = 7.6 Hz, 1H), 7.92 (d, *J* = 8.0 Hz, 1H), 7.87 (s, 1H), 7.73–7.56 (m, 4H), 7.52 (d, *J* = 8.0 Hz, 2H), 7.40–7.33 (m, 1H), 6.60 (s, 1H). ^13^C NMR (101 MHz, DMSO) δ167.7, 160.2, 155.2, 154.1, 140.4, 136.0, 132.8, 131.8, 130.1, 129.9, 128.6, 128.1, 127.5, 127.3, 125.2, 119.0, 117.5, 115.7. The measured weight of CD1 (342.0897) matched the calculated weight (342.0896).

#### 2.6.2 Preparation of 2-[4-(3-Carboxy-4-hydroxy-phenyl)-3-hydroxy-phenyl]­dodecanedioic Acid (FAD1)

##### 2.6.2.1 **Step 1:** Preparation of Methyl 2-(4-Bromo-3-methoxy-phenyl) acetate

A mixture of 2-(4-bromo-3-methoxy-phenyl)­acetic acid (2.4 g, 9.79 mmol) and SOCl_2_ (1.17 g, 9.79 mmol) in MeOH (30 mL) was degassed and purged with N_2_ three times, and then the mixture was stirred at 80 °C for 3 h under N_2_ atmosphere. TLC (PE/EA = 3:1, UV) showed the starting material (*R*_f_ = 0.5) was consumed completely, and one new spot (*R*_f_ = 0.6) was formed. The reaction mixture was quenched by 5% of aqueous Na_2_CO_3_ (20 mL) and extracted with EA (20 mL × 2). The combined organic layers were washed with H_2_O (10 mL × 2), dried over Na_2_SO_4_, filtered, and concentrated under reduced pressure to give a crude product. The crude product was purified by column (PE/EA = 3:1) to afford methyl 2-(4-bromo-3-methoxy-phenyl)­acetate (2.5 g, 98.53% yield) as a yellow oil. ^1^H NMR (400 MHz, CHLOROFORM-*d*) δ 7.48 (d, *J* = 8.0 Hz, 1H), 6.84 (d, *J* = 1.6 Hz, 1H), 6.76 (dd, *J* = 1.6, 8.0 Hz, 1H), 3.91 (s, 3H), 3.71 (s, 3H), 3.60 (s, 2H).

##### 2.6.2.2 **Step 2:** Preparation of Dimethyl 2-(4-bromo-3-methoxy-phenyl) dodecanedioate

To a solution of methyl 2-(4-bromo-3-methoxy-phenyl) acetate (3.25 g, 12.54 mmol) in DMF (40 mL), NaH (652.21 mg, 16.31 mmol, 60% purity) was added. The mixture was stirred for 1 h before adding methyl 10-bromodecanoate (2.66 g, 10.03 mmol) in DMF (10 mL) by a syringe at 0 °C. After stirring at 25 °C for 2 h under N_2_ atmosphere, LCMS showed 41% peak with desired mass. The reaction mixture was quenched by NH_4_Cl (20 mL) and extracted with ethyl acetate (20 mL × 2). The combined organic layers were washed with brine (20 mL × 2), dried over Na_2_SO_4_, filtered, and concentrated under reduced pressure to give a residue. The resulting residue was purified by MPLC (450 g Flash Column Welch Ultimate XB_C18 20–40 μm; 120 A, Solvent for sample dissolution about 2.00 g of sample dissolved in 30 mL of MeCN, 100 mL/min, MeCN/H_2_O) to afford dimethyl 2-(4-bromo-3-methoxy-phenyl) dodecanedioate (2.2 g, 39.56% yield) as a yellow oil. ^1^H NMR (400 MHz, CHLOROFORM-*d*) δ 7.46 (d, *J* = 8.0 Hz, 1H), 6.88–6.84 (m, 1H), 6.78 (dd, *J* = 2.0, 8.0 Hz, 1H), 3.90 (s, 3H), 3.67 (s, 6H), 3.49 (t, *J* = 7.6 Hz, 1H), 2.30 (t, *J* = 7.6 Hz, 2H), 2.12–1.69 (m, 2H), 1.65–1.60 (m, 2H), 1.30–1.20 (m, 12H).

##### 2.6.2.3 **Step 3:** Preparation of Dimethyl 2-[4-(4-hydroxy-3-methoxycarbonyl-phenyl)-3-methoxy-phenyl] dodecanedioate

A mixture of dimethyl 2-(4-bromo-3-methoxy-phenyl) dodecanedioate (1.35 g, 3.04 mmol), methyl 2-hydroxy-5-(4,4,5,5-tetramethyl-1,3,2-dioxaborolan-2-yl)­benzoate (931.48 mg, 3.35 mmol), Pd­(dppf)­Cl_2_.CH_2_Cl_2_ (248.66 mg, 0.304 mmol), Na_2_CO_3_ (968.17 mg, 9.13 mmol) in dioxane (20 mL) and H_2_O (5 mL) was degassed and purged with N_2_ three times, and then the mixture was stirred at 90 °C for 2 h under N_2_ atmosphere. LCMS showed 62% peak with desired mass. The reaction mixture was poured into water (25 mL) and extracted with ethyl acetate (25 mL × 2). The combined organic layers were washed with brine (25 mL), dried over Na_2_SO_4_, filtered, and concentrated under reduced pressure to give a residue. The residue was purified by prep-HPLC (column: Phenomenex Luna C18 150 mm × 25 mm × 10 μm; mobile phase: [H_2_O­(0.225% FA)-ACN]; gradient:63%–93% B over 10.0 min) to afford dimethyl 2-[4-(4-hydroxy-3-methoxycarbonyl-phenyl)-3-methoxy-phenyl] dodecanedioate (1 g, 63.82% yield) as a brown oil. ^1^H NMR (400 MHz, CHLOROFORM-*d*) δ 10.77 (s, 1H), 7.98 (d, *J* = 2.0 Hz, 1H), 7.65 (dd, *J* = 2.0, 8.4 Hz, 1H), 7.23 (d, *J* = 7.6 Hz, 1H), 7.02 (d, *J* = 8.8 Hz, 1H), 6.95 (d, *J* = 7.6 Hz, 1H), 6.92 (s, 1H), 3.95 (s, 3H), 3.83 (s, 3H), 3.68 (d, *J* = 10 Hz, 6H), 3.56 (t, *J* = 7.6 Hz, 1H), 2.30 (t, *J* = 7.6 Hz, 2H), 2.18–1.71 (m, 2H), 1.66–1.60 (m, 2H), 1.28 (br s, 12H).

##### 2.6.2.4 **Step 4:** Preparation of 2-[4-(3-Carboxy-4-hydroxy-phenyl)-3-hydroxy-phenyl] dodecanedioic Acid

A solution of dimethyl 2-[4-(4-hydroxy-3-methoxycarbonyl-phenyl)-3-methoxy-phenyl] dodecanedioate (300 mg, 0.582 mmol) in DCM (10 mL) was degassed and purged with N_2_ three times. The solution was then cooled to 0 °C and maintained under a nitrogen atmosphere. BBr_3_ (2 M, 1.17 mL) was added dropwise to the solution at 0 °C over a period of 10 min. The reaction mixture was stirred at 25 °C for 3 h under N_2_ atmosphere. LCMS showed 62% peak with desired mass. The reaction mixture was quenched by H_2_O (5 mL) at 0 °C, extracted with DCM (10 mL × 2). The combined organic layers were washed with brine (10 mL), dried over Na_2_SO_4_, filtered, and concentrated under reduced pressure to give a residue. The residue was purified by prep-HPLC (column: Phenomenex Luna C18 150 mm × 25 mm × 10 μm; mobile phase: [H_2_O­(0.225% FA)-ACN]; gradient:33%–63% B over 10.0 min) to afford 2-[4-(3-carboxy-4-hydroxy-phenyl)-3-hydroxy-phenyl] dodecanedioic acid (90.1 mg, 33.71% yield) as a white solid. ^1^H NMR (400 MHz, DMSO-*d*_6_) δ 12.81–11.36 (m, 2H), 9.56 (s, 1H), 7.98 (d, *J* = 2.0 Hz, 1H), 7.68 (dd, *J* = 2.0, 8.4 Hz, 1H), 7.18 (d, *J* = 7.6 Hz, 1H), 6.97 (d, *J* = 8.4 Hz, 1H), 6.88 (s, 1H), 6.78 (d, *J* = 7.6 Hz, 1H), 3.42–3.38 (m, 1H), 2.17 (t, *J* = 7.6 Hz, 2H), 1.98–1.82 (m, 1H), 1.69–1.52 (m, 1H), 1.47 (br t, *J* = 6.4 Hz, 2H), 1.22 (br s, 12H). ^13^C NMR (101 MHz, DMSO) δ174.8, 174.5, 172.0, 159.9, 154.1, 140.1, 136.2, 130.8, 130.5, 129.8, 129.1, 123.0, 119.3, 116.7, 115.0, 112.7, 50.6, 40.1, 39.7, 39.7, 39.5, 34.0, 33.7, 28.8, 28.6, 24.5. The measured weight of FAD1 (458.1942) matched the calculated weight (458.1943).

### 2.7 Surface Plasmon Resonance (SPR) Assays

Every protein was immobilized on the CM5 chip (GE) by the method of amine coupling. Conditions for immobilization of protein (or peptide) contained several parameters as follows: surface activation and ligand attachment were performed at 25 °C; specified contact time was 420 s; the concentration of protein was 50–80 μg/mL (every protein was dissolved in 10 mM acetate, pH = 4.5 or 5.0, according to the results of immobilization pH scouting); the aqueous buffer of small-molecule ligands was 1× PBS with 5% DMSO. Other chemicals required for immobilization included 50 mM NaOH solution, ethanolamine, EDC, and NHS. The regeneration scouting contained several parameters as follows: number of regenerations was one; the Prime before run option was selected; contact time was 60 s; stabilization period was 5 s; number of conditions was 3 s; and number of cycles for each condition was five. The regeneration solution for regenerating protein was 10 mM glycine-HCl solution (pH = 3.0 or 3.5). The mode of multicycle kinetics with a 1:1 fit was selected to perform experiments. Different concentrations of ligands were used for analyzing the binding affinities to proteins. The buffer and flow cell-interaction effects have been subtracted from the binding curves.

### 2.8 Coagulation Assessment

C57BL/6 mice were randomly divided into three groups (*n* = 3 per group): control (vehicle-treated), warfarin-treated (5 mg/kg, i.p.), and coumarin derivative-treated (5 mg/kg, i.p.). The test compounds were freshly prepared in saline with 5% DMSO and administered via an intraperitoneal injection. Exactly 30 min after dosing, mice were anesthetized (with isoflurane), and blood was collected via cardiac puncture into prechilled sodium citrate-coated tubes. For coagulation time measurement, 1 drop of blood from every group was immediately transferred onto a sterile glass Petri dish, and a timer was started simultaneously. Coagulation was monitored visually by observing the blood droplet, and coagulation time was recorded as the time (in seconds) taken for the blood to form a stable, nonflowing clot. Group differences in the clotting time were analyzed statistically.

### 2.9 Molecular Docking

The molecular docking simulations were carried out to understand the binding of CD1, HC, DA, and FAD1 to HSA. The X-ray crystal structure of HSA (PDB ID: 1H9Z)^18^ with warfarin was downloaded from the Protein Data Bank (http://www.pdb.org) and used for the molecular docking. The ligand preparation as well as the protein structure preparations, including disulfide bonds and hydrogen atoms addition, were performed by Maestro in Schrödinger (version 11.6.013, Schrödinger, LLC, New York, NY, 2018). The ligands CD1, HC, DA, and FAD1 binding to HSA were docked into the warfarin and myristic acid (MYR) binding sites of HSA using Glide SP module in Schrödinger. For the docking of CD1 and HC to the warfarin binding site of HSA, the grid was defined using a 20 Å box centered on the center mass of CD1. For the docking of DA and FAD1 to the MYR binding site of HSA, the grid was defined using a 20 Å box centered on the center mass of MYR. All other parameters were kept as default. The PyMOL software (DeLano Scientific, Palo Alto, CA, USA) was used to draw the 3D structures of corresponding binding modes of the HC-DA or CD1-FAD1 binding complexes.

### 2.10 Molecular Dynamics (MD) Simulations

The molecular dynamics (MD) simulations were performed for the HC-DA or CD1-FAD1 binding complexes of HSA by AMBER18 ^19^ software to investigate the binding stability and free energy of HC, CD1, DA, and FAD1 to HSA. The partial charges of HC, CD1, DA, and FAD were fitted with B3LYP/def2TZVP^20^ calculations using the restrained electrostatic potential (RESP) module^21^ in the antechamber package of AMBER18.^19^ The general Amber force field (GAFF2) parameters of HC, CD1, DA, and FAD1, including bond, angle, torsion and van der Waals (vdW) terms, were also obtained by the antechamber module.^22,23^ However, the ff19SB force field^24^ was employed for the HSA protein. The protonation states of the ionizable residues of the HSA protein were determined at pH 7 based on p*K*_a_ calculations via both PROPKA^25^ and H++^26^ programs, and the local hydrogen-bonding network was taken into account when the two programs disagreed. Each system of HC-DA or CD1-FAD1 binding complexes was solvated into explicit TIP3P water^25^ molecules using a cubic box with a 15 Å buffer distance between the box wall and its nearest solute atom, and 10 Na^+^ ions were added to neutralize the charge. The energy minimizations and equilibration MD simulations were also following the same sophisticated protocol as in our previous studies.^26^ First, the system was minimized with 1000 steps of steepest descent, followed by 1000-cycle conjugate gradient minimization by restraining heavy atoms of the solvated system (with a restraint force constant of 5 kcal·mol^–1^·Å^–2^) and minimized with the other 1000 steps of steepest descent by restraining the HSA complex. Then, the system was minimized by 1000 steepest descent steps with restraining of backbone atoms. Finally, the system was minimized by 5000 steepest descent steps with no restraining. After Four-step minimization, the system was equilibrated with a 200 ps NVT MD simulation (*T* = 10 K) followed by another 200 ps NPT (*P* = 1 atm) MD simulation with 25 kcal·mol^–1^·Å^–2^ restraint force. Then, the system was heated up from 10 to 310 K with a 50 ps NVT simulation, and equilibrated with a 50 ps NPT simulation, during which the restraint force constant was reduced to 2 kcal·mol^–1^·Å^–2^. Next, one 200 ps NVT equilibration simulations as performed with a constant restraint force constant of 2 kcal·mol^–1^·Å^–2^. Finally, one 100 ns NPT production simulation with 2 kcal·mol^–1^·Å^–2^ restraint was carried out. In all MD simulations, the long-range electrostatic interactions were treated with the particle mesh Ewald (PME)^27^ method, an 8 Å cutoff was used for both vdW and short-range PME interactions, and a time step of 1 fs and the SHAKE algorithm^28^ were used. The analysis of the MD trajectories was performed by cpptraj module in AMBER18.

The binding free energies of HC-, CD1-, DA-, and FAD1-HSA complexes were calculated using the MM-GBSA module implemented in Amber18^33–35^. A single-trajectory protocol was employed, in which snapshots extracted from the MD trajectory of each complex were used to evaluate the free energies of the complex, receptor, and ligand. For each system, 1000 snapshots were extracted from the 100 ns MD trajectory at an interval of 0.1 ns. The polar solvation contribution was estimated using the generalized Born (GB) model with igb = 5, with a salt concentration of 0.15 M. In addition, per-residue free energy decomposition was performed by using idecomp = 1.

## 3 Results and Discussion

### 3.1 Random Forest Classifier as a Surrogate Scorer for HSA Binders

To establish a reliable predictor for downstream optimization, we first constructed a Random Forest classifier trained on a curated data set to distinguish HSA binders (KD or *K*_i_ < 1 mM, i.e., measurable binding) from nonbinders. This cutoff was used to balance sample size and label noise in public binding data sets rather than to imply high affinity (Figure 3a). The model’s performance was rigorously evaluated using a 5-fold cross-validation scheme and an independent test set. The classifier achieved strong discriminative ability, with an AUC-ROC of 0.93, an accuracy of 0.89, a precision of 1.00, and a recall of 0.48 on the test set. As illustrated in Figure 3b, the classifier achieved a high predictive accuracy on the test set in 5-fold cross-validation, demonstrating its robustness in identifying potential albumin-binding motifs.

**Figure 3:**
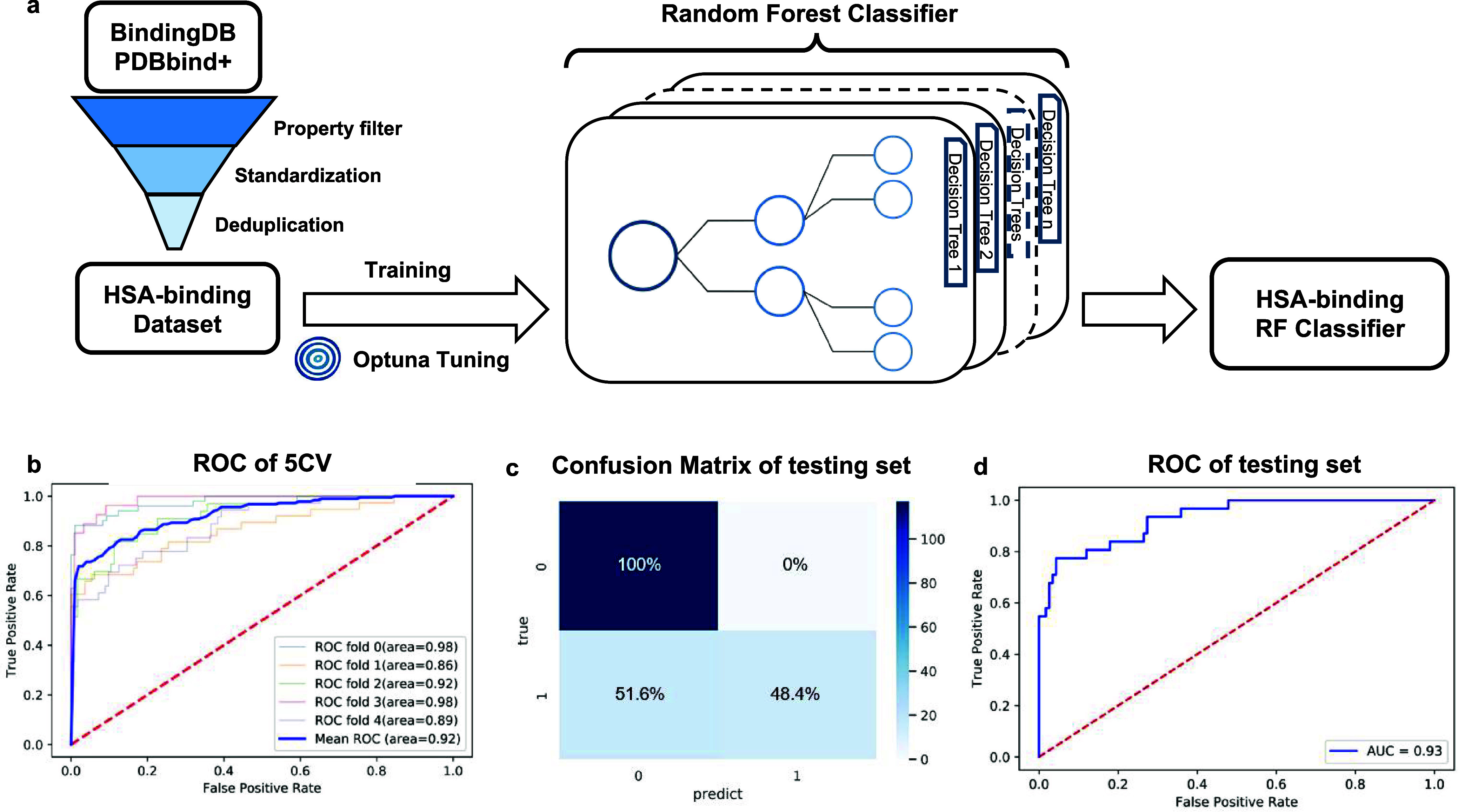
The schematic diagram and the result of HSA-binding random forest classifier for scoring function. (a) Schematic diagram of random forest classifier model. (b) 5-fold cross-validation ROC curve during model training. (c) Confusion matrix of the trained HSA-binding RF classifier on testing set. (d) ROC curve of the trained HSA-binding RF classifier on testing set. Note: HSA: human serum albumin; RF: random forest; ROC: Receiver operating characteristic; CV: cross-validation.

Beyond accuracy, the model exhibited a Matthews correlation coefficient (MCC) of 0.65 and a Cohen’s Kappa score of 0.60, indicating a substantial agreement between predicted and actual classifications even within an imbalanced data set. Although the F1-score was 0.65, this reflects a strategic trade-off to maximize specificity, ensuring that false positives are minimized during the generative phase. The confusion matrix and ROC curves further confirm the model’s discriminatory power (Figure 3c,d). Given the imbalanced nature of the data set, we additionally evaluated the model using a Precision-Recall curve (Figure S11), which yielded an average precision (AP) of 0.879. Collectively, these results validate the classifier’s suitability as a scoring function for the subsequent generative deep learning workflow.

### 3.2 Reinforcement Learning-Guided Scaffold Decoration Expands Coumarin Analog Space

Leveraging the validated Random Forest model as a scoring function, we employed the Libinvent deep generative framework to generate a coumarin analogue space while preserving the core chemotype (Figure 4a). The generative process was driven by a reinforcement learning objective to maximize the predicted HSA binding probability. As shown in the training trajectory (Figure 4b), the average reward score exhibited a rapid increase during the initial phase and stabilized at a plateau around epoch 300. Distributions of the predicted binding probability score of the generated CD library are plotted in Figure S12. Structural analysis of the generated library revealed a marked enrichment in aromatic ring systems (Figure 4C). This prevalence suggests that the model implicitly learned the importance of pi–pi stacking and hydrophobic interactions in albumin binding, favoring phenyl ring insertions to enhance stability and target affinity. The resulting library represents a focused yet chemically diverse expansion of the original coumarin scaffold, tailored specifically for HSA interaction.

**Figure 4:**
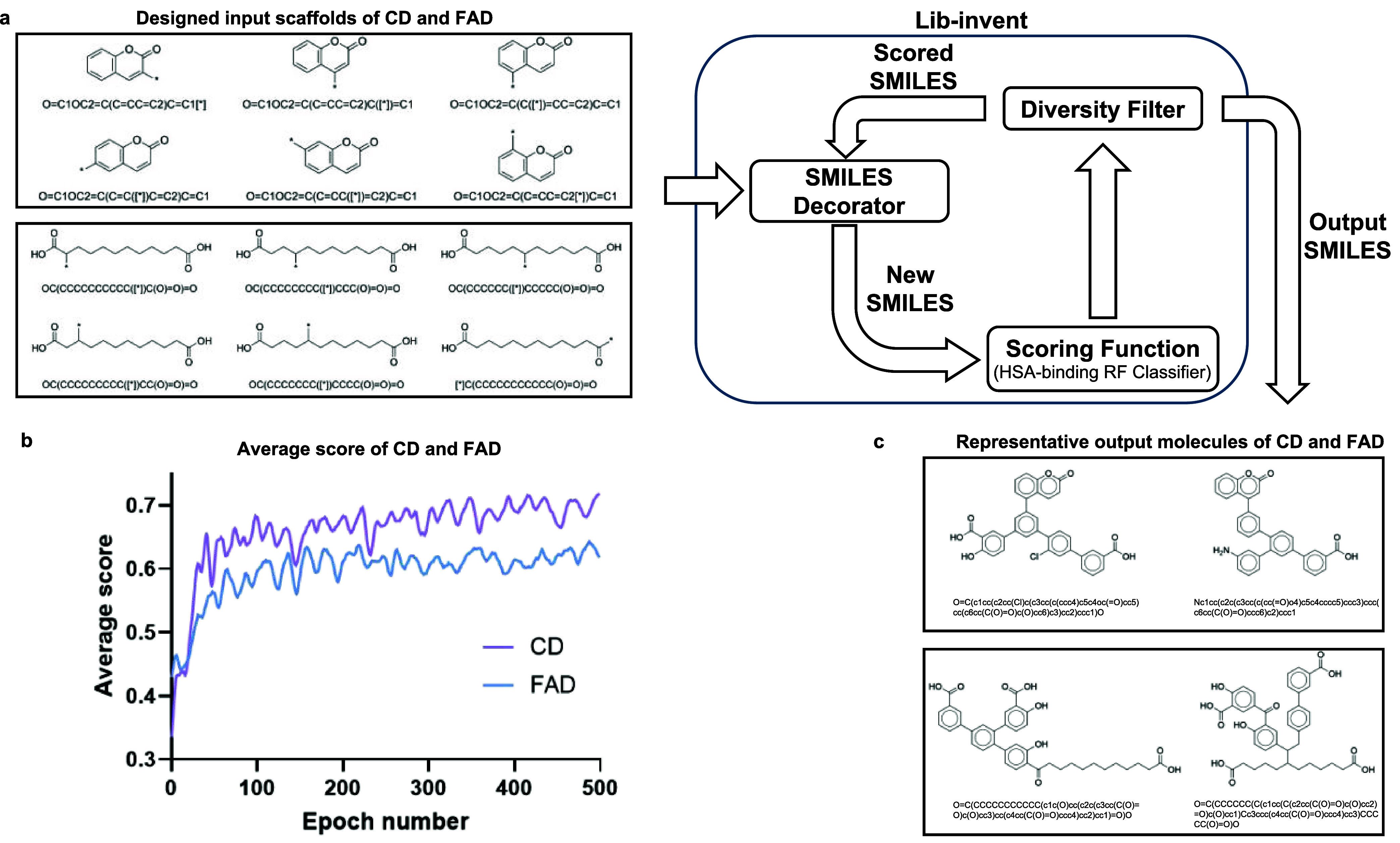
The Lib-invent model training of CD and FAD. (a) The designed input scaffolds and the schematic diagram of Lib-invent model for CD and FAD. (b) The average score of generated molecules during the Lib-invent model training. (c) The representative output molecules of CD and FAD.

### 3.3 Reinforcement Learning-Guided Scaffold Decoration Expands Fatty Acid Analog Space

In parallel, we optimized the fatty acid component by applying the same reinforcement learning-guided scaffold decoration strategy to generate fatty acid-derived (FAD) analogs aimed at engaging fatty acid-binding pockets on HSA (Figure 4a). The optimization trajectory for the FAD scaffold was notably faster. The reward score increased rapidly and reached a convergence plateau at approximately epoch 100 (Figure 4b). This rapid convergence likely reflects the distinct structural simplicity of fatty acid analogues compared with coumarin derivatives. Distributions of the predicted binding probability score of the generated FAD library are plotted in Figure S12. This indicated that the generated molecules are generally predicted to have favorable HSA binding, with the coumarin series scoring slightly higher on average. The top-ranking FAD candidates featured structural modifications, such as bulky hydrophobic appendages and aromatic substituents onto the fatty acid chain, which were predicted to enhance hydrophobic contacts within the albumin binding sites (Figure 4c). By running these two parallel design streams, we ensured coverage of different binding modalities on the albumin surface.

### 3.4 Filtering and Diversity Clustering Prioritize Representative Candidates CD1 and FAD1

The statistics of the property of the generated CD and FAD libraries is shown in Table S3. The corresponding similarity plots to train set and to scaffold molecules for the generated libraries are shown in Figure S13. These data collectively demonstrate that the generative workflow produced a large, chemically valid, diverse, and predominantly novel set of molecules that remain anchored around the desired chemotypes while exploring promising binding-relevant modifications. To select the most promising candidates from the thousands of AI-generated molecules for experimental validation, we implemented a hierarchical virtual screening strategy. Initially, a strict physicochemical filter was applied: only molecules with a molecular weight less than 500 Da and a predicted binding probability score bigger than 0.8 were retained to ensure drug-likeness and high predicted binding probability.

The surviving candidates were then subjected to Butina clustering to analyze the structural similarity and remove redundancy. Visualization of the chemical space using t-distributed stochastic neighbor embedding (t-SNE) revealed distinct clusters for both CD and FAD series, confirming the structural diversity of the generated library (Figure 5). To evaluate the synthesizability, the synthetic accessibility score (SAScore) was calculated (Table S1). From the most populous and high-scoring clusters, we identified the centroid molecules, designated as CD1 (SAScore = 1.94) and FAD1 (SAScore = 2.89), as representative candidates for chemical synthesis and biophysical validation.

**Figure 5:**
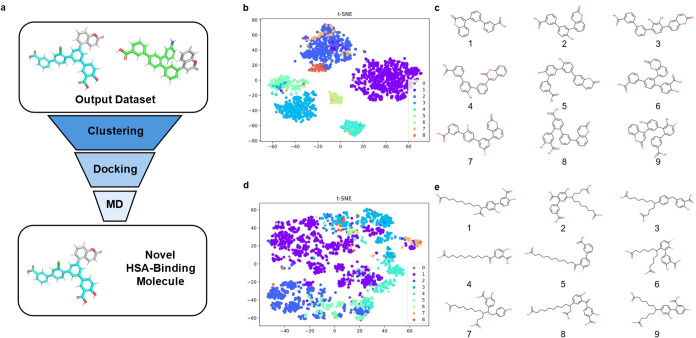
Butina clustering result of output data set. (a) The schematic diagram of downstream screening of the output molecules. (b) t-SNE plot of the Butina clustering results of CD. (c) Structures of the centroid representatives for the top 9 clusters of CD sorted by cluster size. (d) t-SNE plot of the Butina clustering results of FAD. (e) Structures of the centroid representatives for the top 9 clusters of FAD sorted by cluster size.

### 3.5 Chemical Synthesis and Characterization of CD1 and FAD1

The identified lead compounds, CD1 and FAD1, were synthesized following a custom-designed synthetic route tailored to their specific structural features (Figures S1 and S3). The chemical identity and purity of the final products were unequivocally confirmed via high-resolution mass spectrometry (MS) and nuclear magnetic resonance (^1^H NMR and ^13^C NMR) spectroscopy. As shown in Figures S2 and S4, the spectral data were consistent with the predicted structures.

### 3.6 Biophysical Validation Confirms Nanomolar HSA Binding and Enhanced Affinity of Optimized Analogs

The binding kinetics and affinity of the synthesized leads to HSA were quantitatively characterized using surface plasmon resonance (SPR). As depicted in Figure 6, both compounds exhibited concentration-dependent binding responses. Notably, the optimized coumarin derivative, CD1, demonstrated an exceptionally high affinity with an equilibrium dissociation constant (KD) of 5.02 nM. The fatty acid derivative, FAD1, also showed strong binding with a KD of 33.2 nM. These nanomolar affinities validated the efficacy of our AI-driven scaffold-focused optimization strategy in identifying superior ligands. Statistics for comparing the properties of the generated CD1, FAD1, and their scaffold molecules (HC and DA) is shown in Table S4.

**Figure 6:**
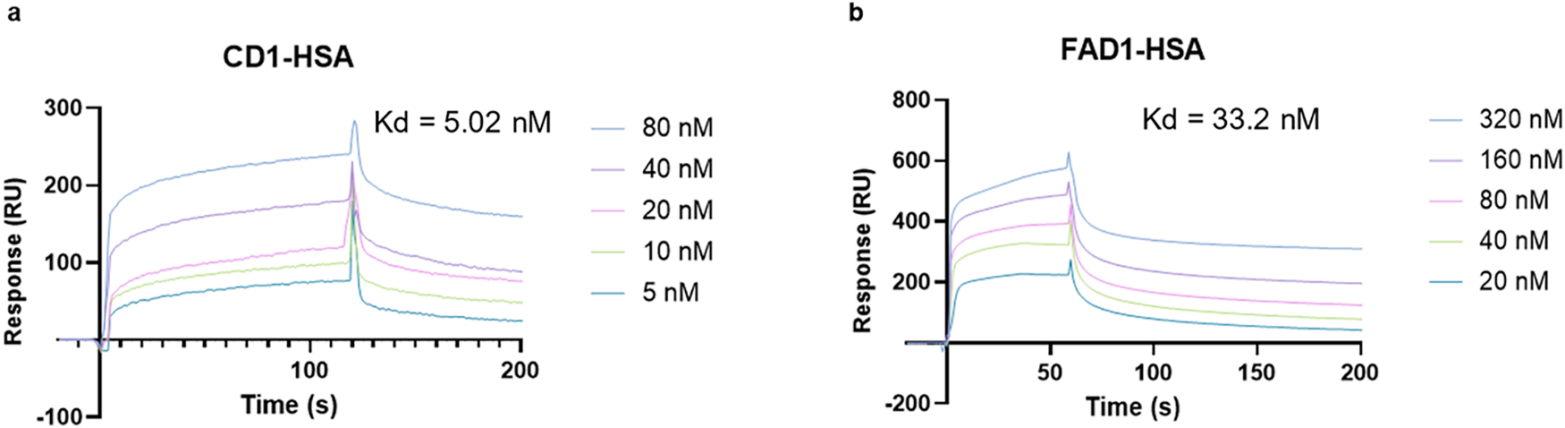
Binding analysis of CD1 and FAD1 to HSA by SPR assay, respectively. (a) SPR analysis of CD1 to HSA. (b) SPR analysis of FAD1 to HSA.

### 3.7 Acute Clotting-Time Assay Shows No Detectable Anticoagulant Activity for CD1

Given that coumarin derivatives (e.g., warfarin) are historically associated with anticoagulant activity, it was critical to assess the safety profile of CD1. We conducted a clotting time assay in C57BL/6 mice to evaluate its potential interference with the coagulation cascade. As shown in Figure S5, unlike warfarin, CD1 did not induce any significant prolongation of the clotting time compared to the control group. This result indicates that the structural modifications introduced by the AI model to enhance albumin binding successfully avoided the pharmacophore responsible for anticoagulant side effects, suggesting a favorable safety window for CD1 as a therapeutic modifier.

### 3.8 Docking and MD Simulations Rationalize Affinity Improvements at Corresponding HSA Sites

To investigate and compare the binding of HC-DA combination and CD1-FAD1 combination to HSA protein, we performed molecular docking of these ligands to HSA protein and carried out molecular dynamics (MD) simulations for the HC-DA or CD1-FAD1 binding complexes of HSA (Figures 7 and S6–S10, and Tables S2). The binding modes through molecular docking shown in Figures 7 and S6–S8 revealed that HC and CD1 occupy the warfarin binding site of HSA (PDB ID: 1H9Z), while DA and FAD1 occupy the five binding sites of myristic acid (MYR). CD1 forms salt-bridge interactions with R218 and R222 of HSA, while HC forms hydrogen-bonding interactions with R222 and K199 of HSA. The binding free energies of HC and CD1 to HSA protein were −21.08 and −38.11 kcal/mol, respectively (Table S2, average binding affinity with two enantiomers of FAD1). Thus, CD1 binds tighter to HSA than HC, consistent with the SPR experiment results that CD1 shows a higher binding affinity than HC. For binding comparison between DA and FAD1, as shown in Figures 7 and S6–S8, FAD1­(R)­3 and FAD1­(S)­3 form additional hydrogen bonds with R484, indicating that FAD1 performs more stable and stronger interactions with the receptor in additional polar branches. The average binding free energies of DA and FAD1 to HSA were −53.47 and −57.73 kcal/mol, respectively. Therefore, FAD1 exhibits a higher binding affinity to HSA than DA, agreeing well with the SPR experiment results.

**Figure 7:**
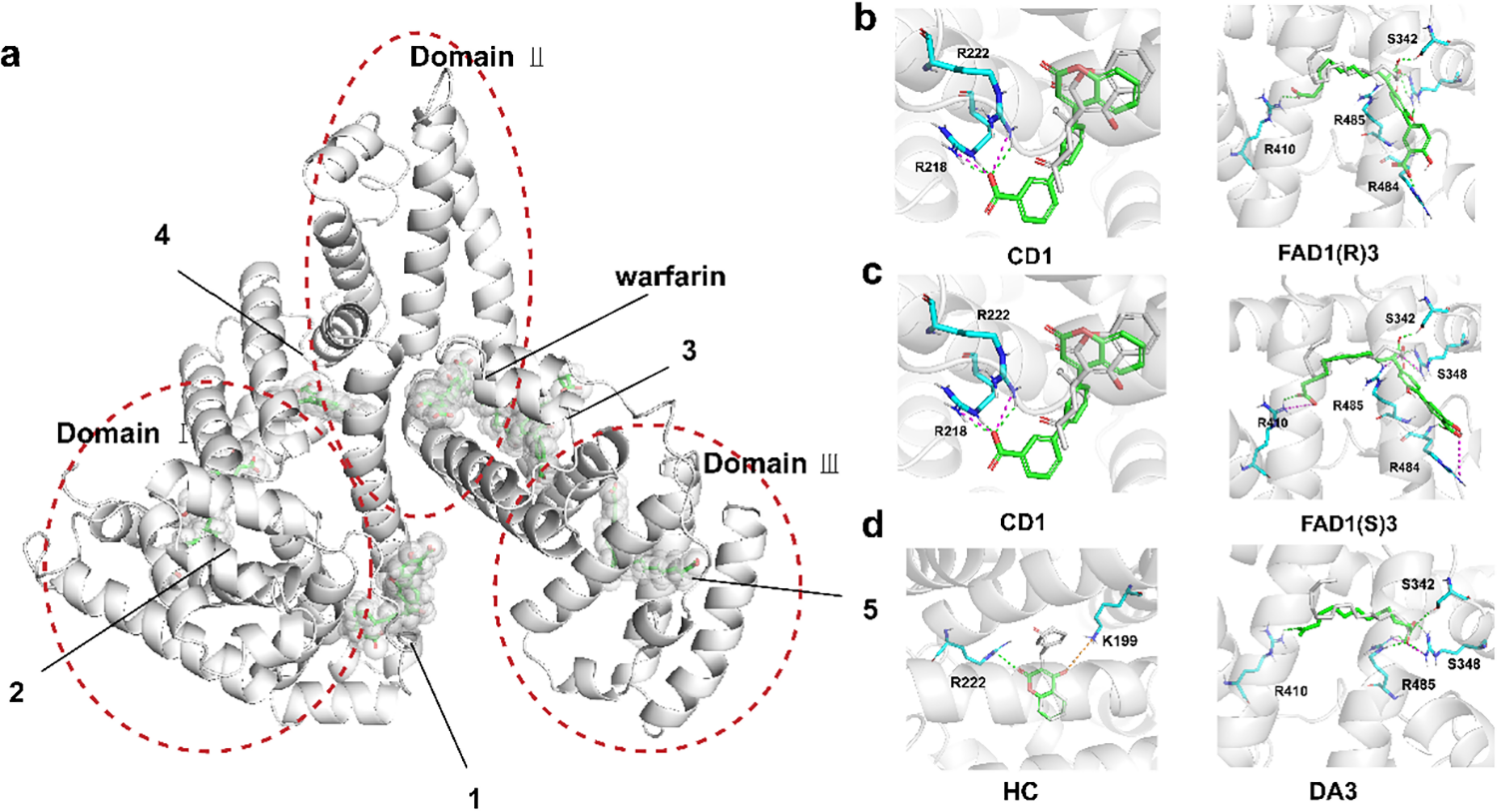
The binding modes of HC, CD1, DA, and FAD1 to HSA. (a) The binding site and domains of HSA binding with warfarin and MYR (PDB ID: 1H9Z). The protein is shown as cartoon and colored white, and the pocket is shown by a gray-colored surface. The sites 1–5 present the binding site of FAD1 and DA. (b–d) The binding mode of HC, CD1, DA, and FAD1 to HSA. HC, CD1, DA, and FAD1 are colored in green, the original ligand is colored in white, and the surrounding residues are colored in blue. The salt-bridge interactions are indicated with orange dashed lines, and the H-bond interaction is indicated with a green dashed line.

### 3.9 Discussion

Optimizing albumin-binding small molecules often relies on empirical iteration and extensive screening, which limit chemical diversity and efficiency. Here we show that a scaffold-focused, AI-guided workflow can systematically expand analog space while preserving the core chemotypes and, importantly, can translate computational enrichment into experimentally validated improvements. A random forest classifier served as an external scorer to guide LibINVENT reinforcement learning, and candidates were prioritized through a selection funnel combining physicochemical constraints, probability thresholding, diversity-aware clustering, and synthetic feasibility assessment. The fact that the workflow converged on CD1 and FAD1 as representative centroid leads that were successfully synthesized and validated indicates that the closed-loop strategy is practically feasible rather than being purely exploratory.

SPR experiments confirmed that the optimized representatives show nanomolar binding to HSA and enhanced affinity compared with the original ligands in the dual combination. Importantly, the direction of affinity improvement observed in SPR is consistent with the computational analysis, supporting internal coherence between wet-lab and dry-lab results. Molecular docking and MD simulations suggested that coumarin derivatives bind to the canonical warfarin site, while fatty acid derivatives engage fatty acid-binding pockets on HSA. For the coumarin component, CD1 was predicted to form additional stabilizing salt-bridge interactions with residues such as R218 and R222, together with more favorable binding energetics compared with HC, which provides a mechanistic rationale that matches the experimental trend. For the fatty acid component, FAD1 exhibited additional stabilizing contacts and more favorable binding energetics relative to those of DA, again aligning with the experimental direction of improvement.

Although docking and MM GBSA-based energetics are not expected to reproduce absolute SPR affinity values, their role here is to provide a consistent mechanistic explanation for why the optimized analogs bind more strongly. This wet–dry agreement increases confidence that the closed-loop optimization did not merely exploit artifacts of the predictive model, but captured chemically meaningful features that translate to measurable binding.

A major safety concern in repurposing coumarin scaffolds for drug delivery is the potential retention of vitamin K antagonist (VKA) activity, which could induce iatrogenic bleeding. Warfarin exerts its anticoagulant effect by inhibiting the vitamin K epoxide reductase complex subunit 1 (VKORC1), a mechanism critically dependent on the acetonylbenzyl moiety at the 3-position of the coumarin ring. Our *in vivo* safety assessment indicated that CD1 does not prolong clotting time 30 min postadministration. The de novo design introduced a linker at the 3-position of the coumarin core, which sterically disrupted the pharmacophore required for VKORC1 binding. Thus, CD1 appears to function as an albumin binder that retains a high affinity for the carrier protein while evading the enzymatic targets associated with coumarin toxicity.

While this work focuses on optimizing and validating the small-molecule components for binding to HSA, our broader motivation is to design a super-long-lasting nucleic acid aptamer drug. We intend to conjugate the optimized dual combination into an aptamer, thereby forming a dual-modified aptamer conjugate with high binding affinity to HSA. In our preliminary experiments, the aptamer conjugated with the CD1–FAD1 combination demonstrated superior affinity HSA compared to the aptamer modified with their parent combination (DA and HC), although these results are not shown in this manuscript. As such, we anticipate that this enhanced binding will translate into a synergistic extension of the conjugated aptamer’s circulation half-life, analogous to the effect observed with the parent combination. To validate this, further pharmacokinetic studies are required to quantify the extent of half-life extension across different doses and administration routes. Subsequently, pharmacodynamic studies will be necessary to determine whether the optimized combination confers measurable efficacy benefits in relevant disease models.

## 4 Conclusions

In this study, we developed a closed-loop, scaffold-focused AI workflow that integrates a machine-learning surrogate scorer with LibINVENT reinforcement learning, followed by multistage prioritization, wet-lab synthesis, and SPR validation. Using this framework, we optimized a synergistic HSA-binding dual combination while preserving the core chemotypes and identified two representative leads, CD1 and FAD1, that exhibit nanomolar HSA binding and improved affinity relative to the parent ligands. Docking and molecular dynamics simulations provided a mechanistic rationale consistent with the experimental trends, and an acute clotting time assay served as a preliminary safety screen for the coumarin analog. Going forward, we will conjugate the optimized CD1–FAD1 combination onto nucleic acid aptamers and systematically evaluate pharmacokinetics across doses and administration routes, as well as pharmacodynamic benefits in relevant disease models.

## Supplementary Material



## Data Availability

All the data underlying this study stem from freely available, public data sources. The data that support the findings of this work have been included in the main text and Supporting Information (SI). The source code is available at https://github.com/Mirakit/Albumin-Binders

## Notes

Designed synthetic routes of CD1 and FAD1 (Figures S1 and S3); NMR and MS spectra of CD1 and FAD1 (Figures S2 and S4); clotting time of CD1 (Figure S5); docking results (Figures S6–S8); predicted binding pocket and binding mode (Figure S9); RMSD (Figure S10); precision-recall curve (Figure S11); score distribution (Figures S12 and S13); effect of positional restraints on ligand stability (Figure S14); SAScore of centroid CD and FAD (Table S1); computational binding free energies (Table S2); Statistics of the property of the generated CD and FAD library (Table S3); and statistics of the property of the generated CD1, FAD1, and their scaffold molecules (Table S4) (PDF)
