# Supplementary material for: Generative Artificial Intelligence Optimization of Albumin Binders: Coumarin and Fatty Acid Derivatives
Source: J Chem Inf Model. 2026 Jun 2;66(12):6910–20. doi: 10.1021/acs.jcim.6c00296 (PMC13292218; doi:10.1021/acs.jcim.6c00296)
Supplement: Supplementary file 1 [file ci6c00296_si_001.pdf]

Supporting Information

Generative artificial intelligence optimization of albumin binders: coumarin and fatty acid derivatives

Yihao Zhang[1,2,#], Qirui Deng[3,#], Xin Yang[1,#], Xinchen Yue[3], Huarui Zhang[2], Shijian, Ding[1], Jinping Lei [3,*], Baoting Zhang[2,*], Sifan Yu[1,4,*], Ge Zhang[1,4,*]

1. *Law Sau Fai Institute for Advancing Translational Medicine in Bone and Joint Diseases (TMBJ), School of Chinese Medicine, Hong Kong Baptist University, Hong Kong SAR, 000000, China*
2. *School of Chinese Medicine, Faculty of Medicine, The Chinese University of Hong Kong, Hong Kong SAR, 000000, China*
3. *School of Pharmaceutical Sciences, Sun Yat-Sen University, Guangzhou 510006, China*
4. *Shenzhen Institute for Research and Continuing Education (IRACE), Hong Kong Baptist University, Shenzhen, 518000, China*

[#] Yihao Zhang, Qirui Deng, and Xin Yang contributed equally to this work.

*Corresponding authors
E-mail: Baoting Zhang (zhangbaoting@cuhk.edu.hk), Jinping Lei (leijp@mail.sysu.edu.cn), Sifan Yu (yusifan123@gmail.com), Ge Zhang (zhangge@hkbu.edu.hk)

Table of Contents

Figure S1. The designed synthetic route of CD1.
Figure S2. NMR and MS spectrum of CD1.
Figure S3. The designed synthetic route of FAD1.
Figure S4. NMR and MS spectrum of FAD1.
Figure S5. The clotting time of CD1.
Figure S6. The docking result of CD1 and FAD1(R) with HSA.
Figure S7. The docking result of CD1 and FAD1(S) with HSA.
Figure S8. The docking result of HC and DA with HSA.
Figure S9. The predicted binding pocket and binding mode of CD1 and HC with HSA.
Figure S10. The root mean square deviation (RMSD) of the protein backbone, ligand, and complex of three system.
Figure S11. The Precision-Recall Curve of the HSA binding classifier.
Figure S12. The score distribution of the generated CD and FAD library.
Figure S13. The score distribution of similarities of the generated CD and FAD libraries to train set and to scaffold molecule, respectively.
Figure S14. Effect of positional restraints on ligand stability during 100 ns MD simulations.
Table S1 The Synthetic Accessibility Score (SAScore) of centroid CD and FAD.
Table S2. The computational binding free energy for HC, CD1, DA and FAD1 on the binding pocket of HSA.
Table S3. Statistics of the property of the generated CD and FAD library.
Table S4. Statistics of the property of the generated CD1, FAD1, and their scaffold molecules (HC and DA), respectively.

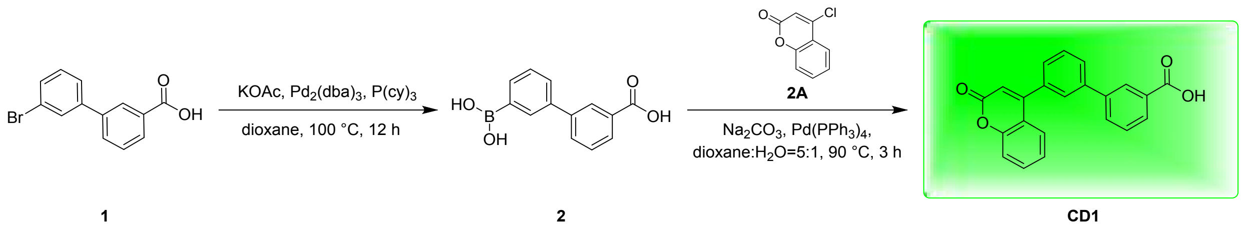

Figure S1. The designed synthetic route of CD1.

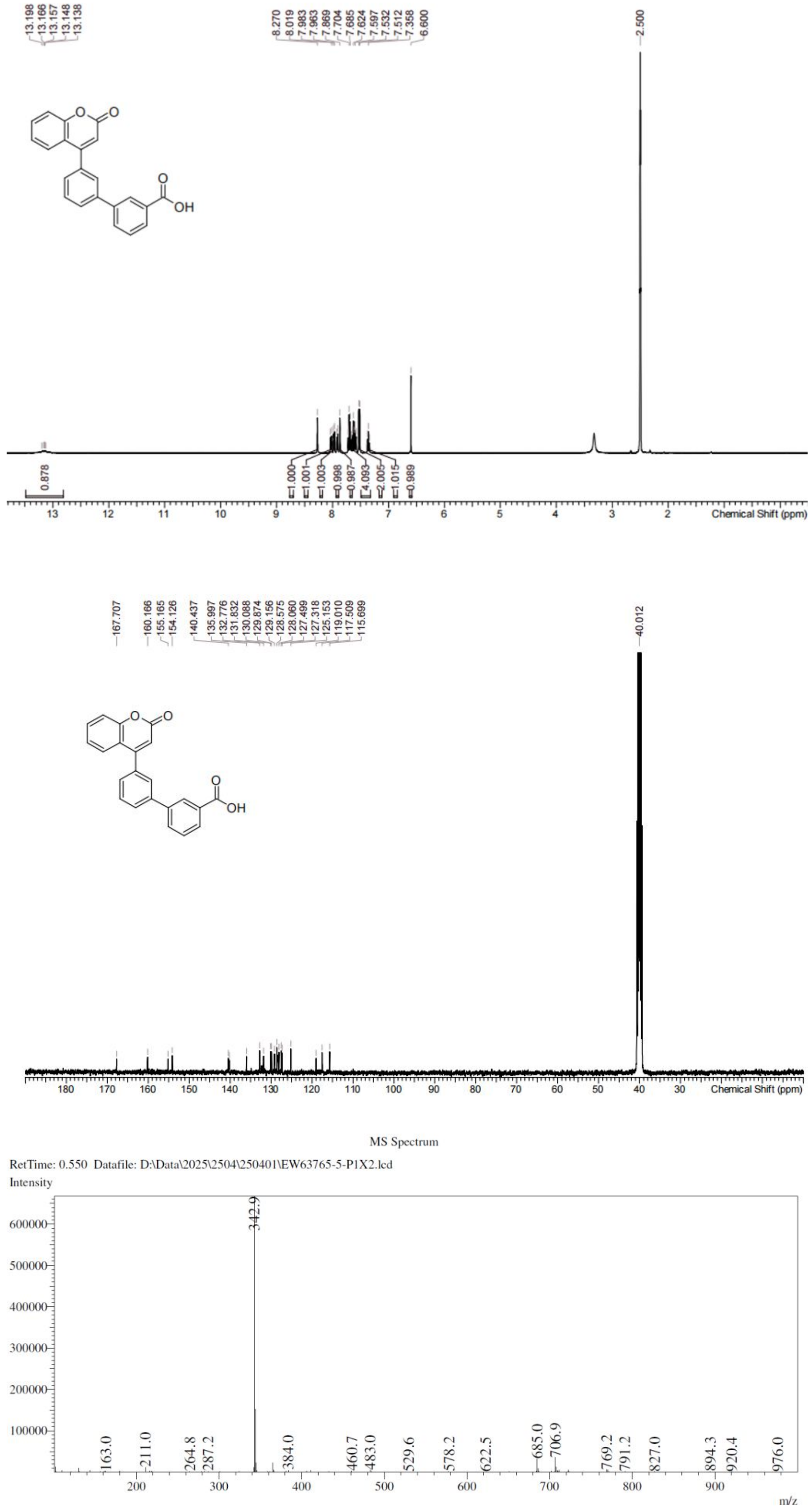

Figure S2. NMR and MS spectrum of CD1.

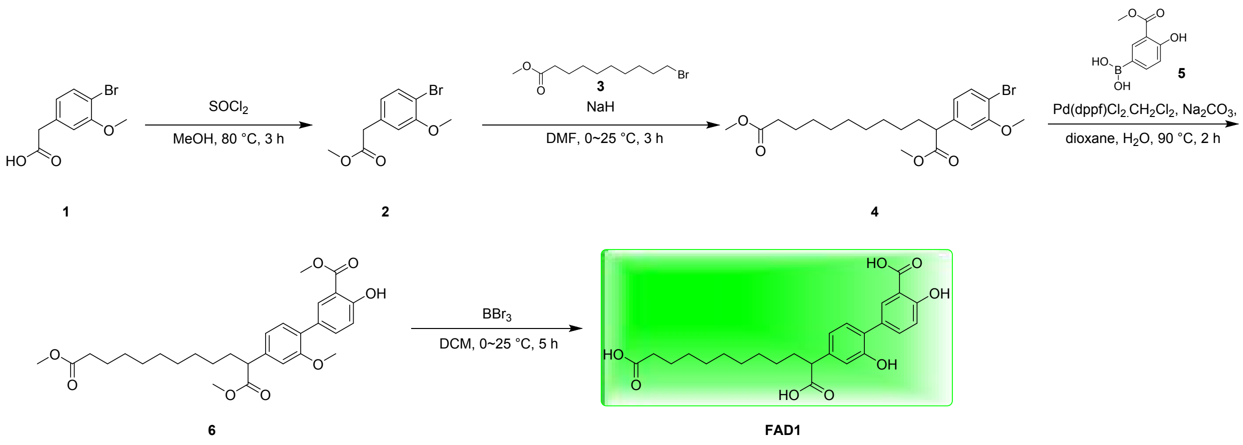

Figure S3. The designed synthetic route of FAD1.

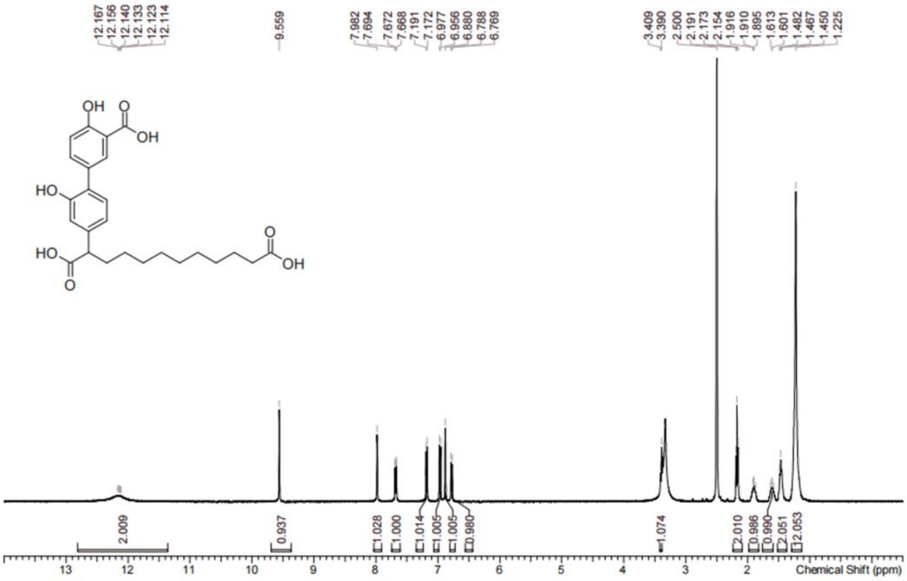

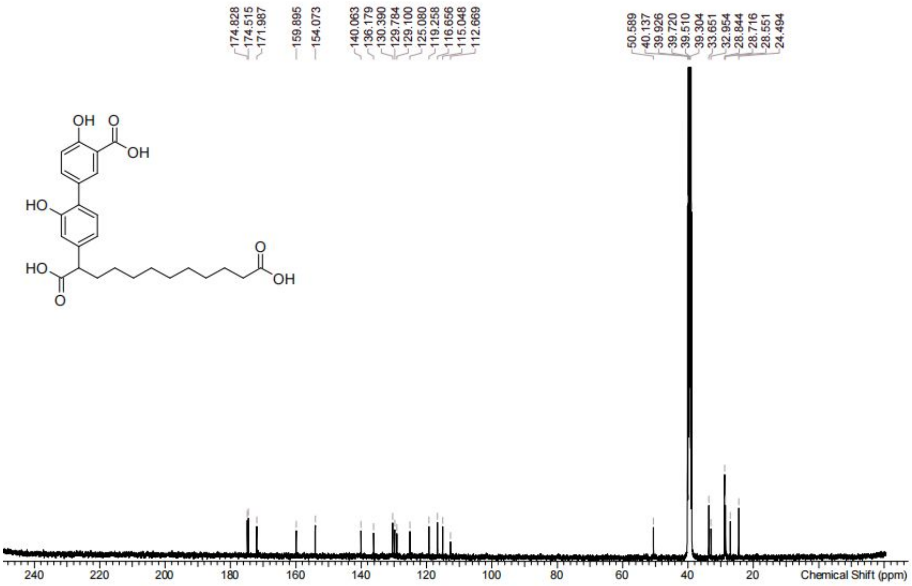

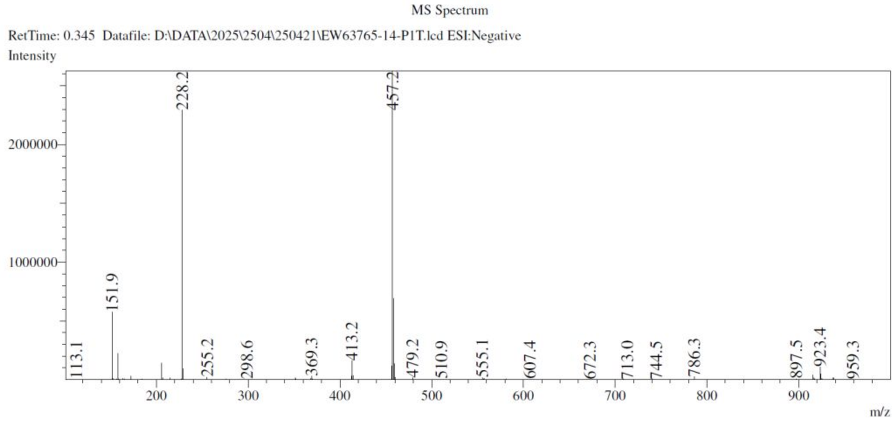

Figure S4. NMR and MS spectrum of FAD1.

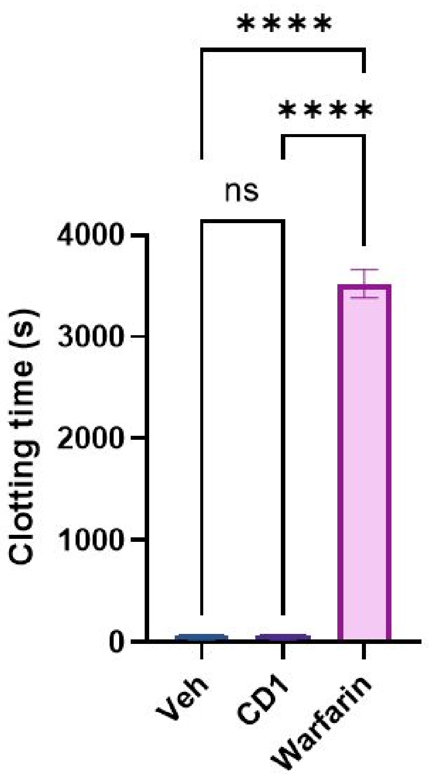

Figure S5. The clotting time of CD1.

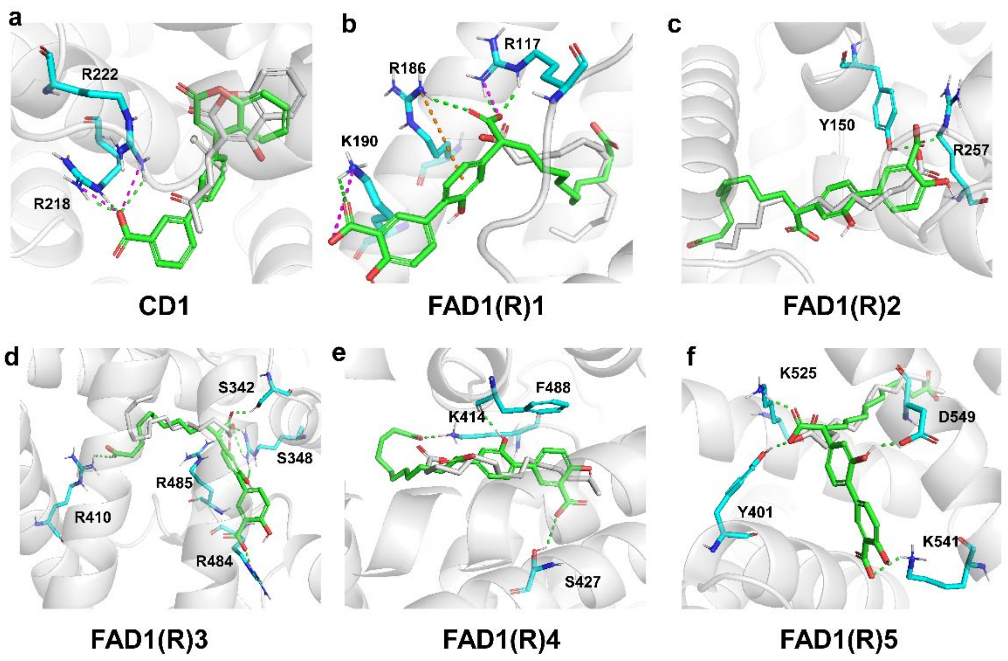

Figure S6. The docking result of CD1 and FAD1(R) with HSA. **a**, the binding pose of CD1 with HSA. **b-f,** the binding pose of FAD1(R) with HSA. CD1 and FAD1 are colored in green and the surrounded residues of HSA is shown in stick and colored in blue. The protein is colored in white.

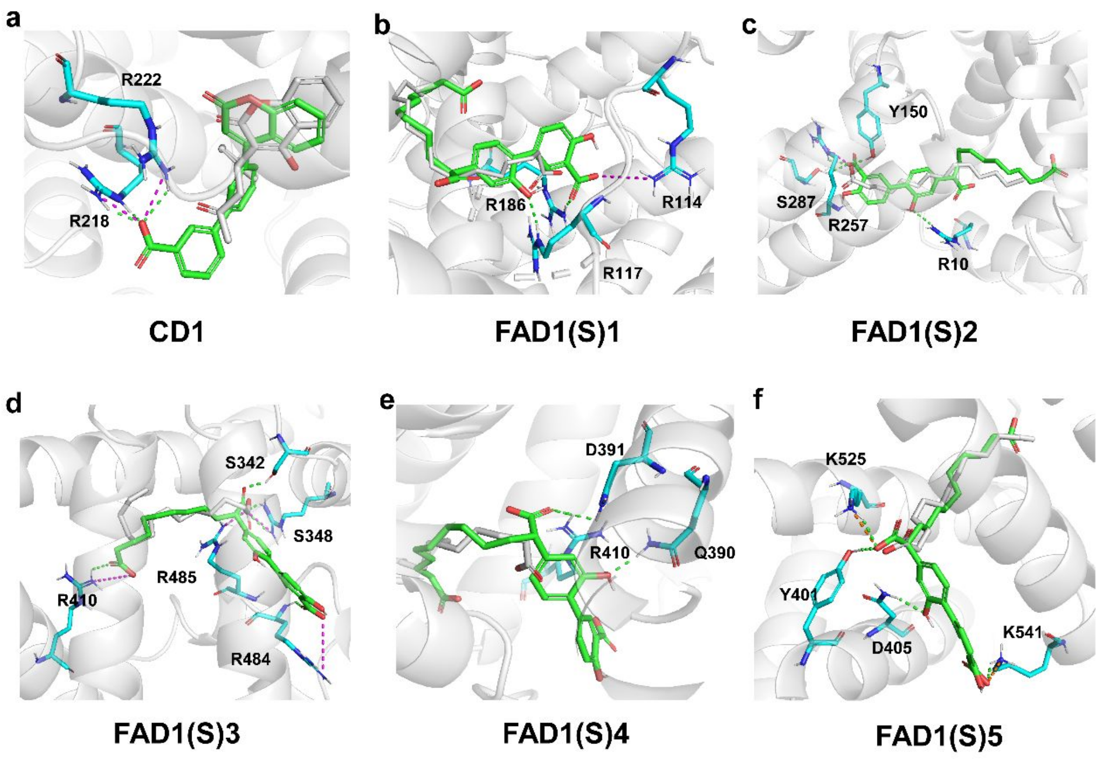

Figure S7. The docking result of CD1 and FAD1(S) with HSA. **a**, the binding pose of CD1 with HSA. **b-f,** the binding pose of FAD1(S) with HSA. CD1 and FAD1 are colored in green and the surrounded residues of HSA is shown in stick and colored in blue. The protein is colored in white.

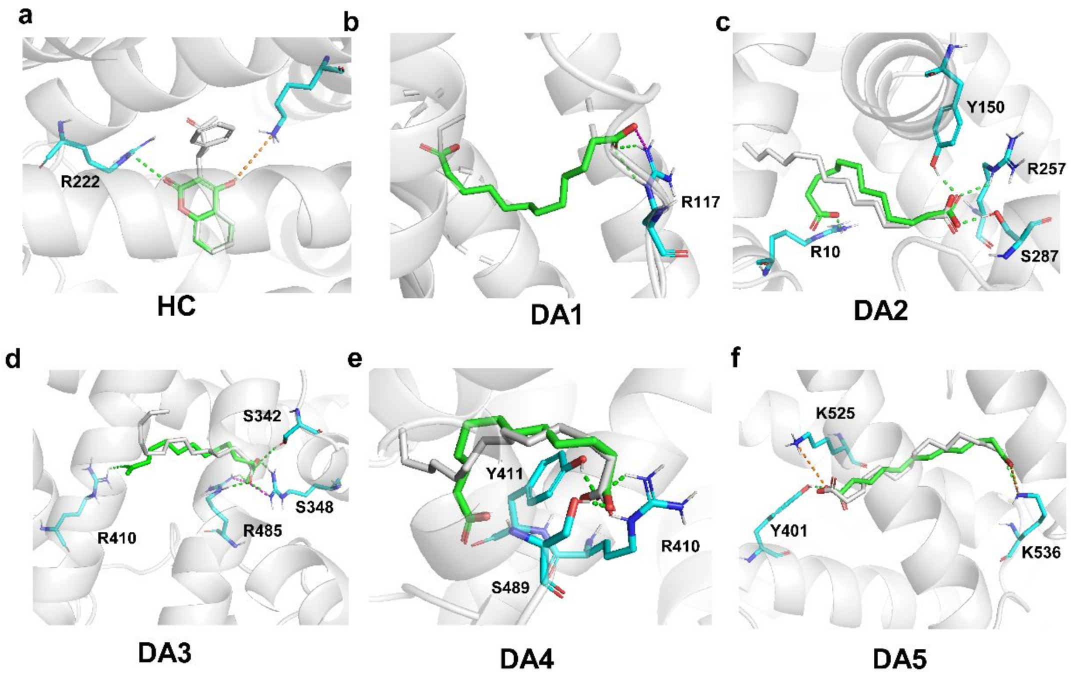

Figure S8. The docking result of HC and DA with HSA. **a**, the binding pose of HC with HSA. **b-f,** the binding pose of DA with HSA. HC and DA are colored in green and the surrounded residues of HSA is shown in stick and colored in blue. The protein is colored in white.

a

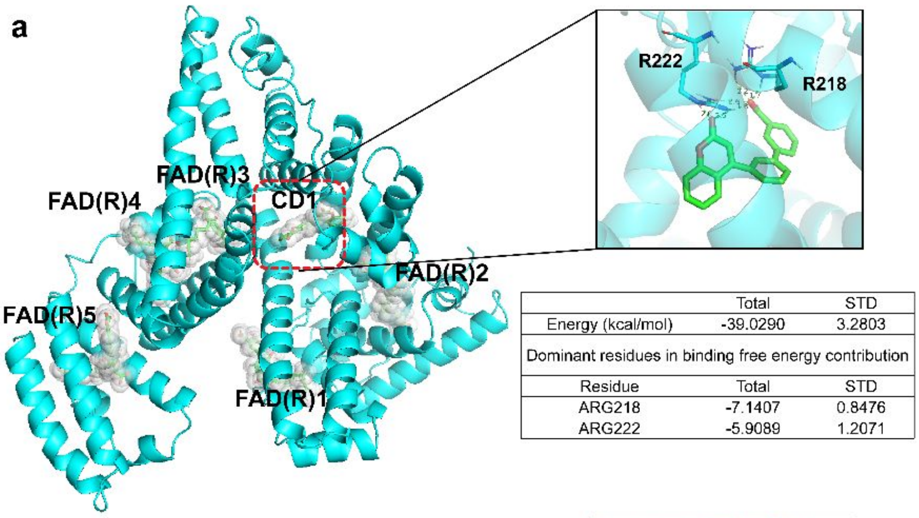

	Total	STD
Energy (kcal/mol)	-39.0290	3.2803
Dominant residues in binding free energy contribution		
Residue	Total	STD
ARG218	-7.1407	0.8476
ARG222	-5.9089	1.2071

b

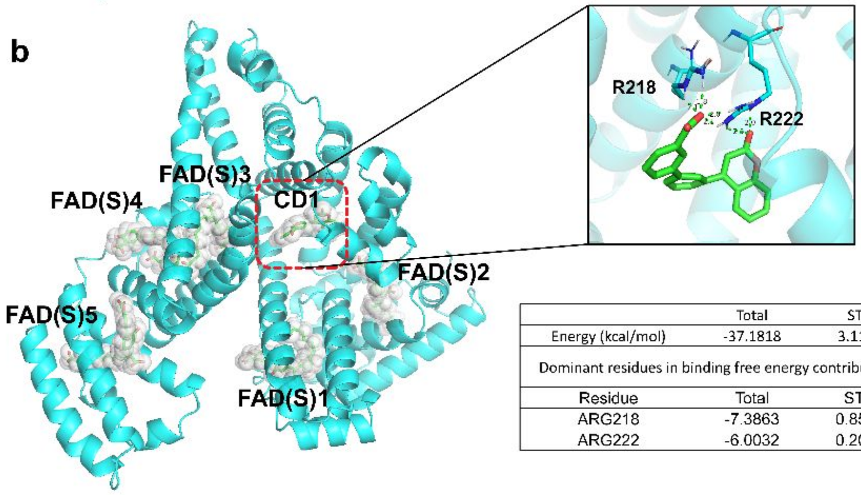

	Total	STD
Energy (kcal/mol)	-37.1818	3.1141
Dominant residues in binding free energy contribution		
Residue	Total	STD
ARG218	-7.3863	0.8567
ARG222	-6.0032	0.2001

c

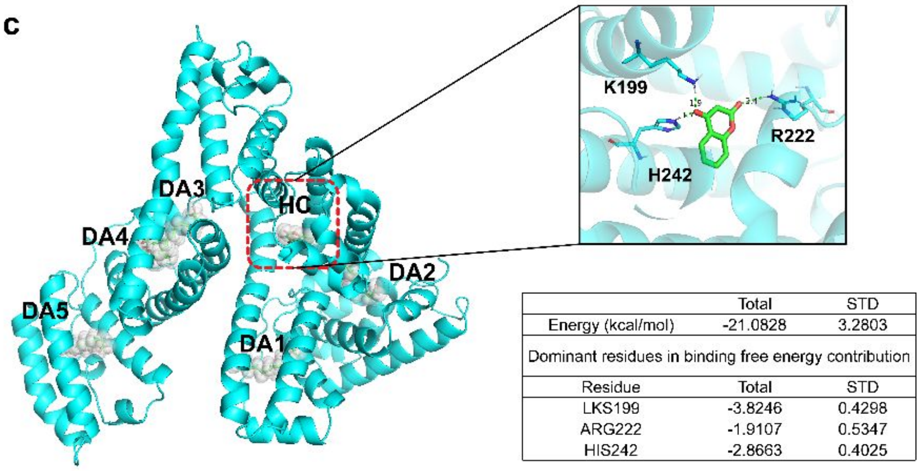

	Total	STD
Energy (kcal/mol)	-21.0828	3.2803
Dominant residues in binding free energy contribution		
Residue	Total	STD
LKS199	-3.8246	0.4298
ARG222	-1.9107	0.5347
HIS242	-2.8663	0.4025

Figure S9. The predicted binding pocket and binding mode of CD1 and HC with HSA. **a**, the binding mode of CD1 with HSA and FAD1(R). **b**, the binding mode of CD1 with HSA and FAD1(R). **c**, the binding mode of HC with HSA and DA. The protein is shown as cartoon and colored in blue, and the pocket is shown by gray colored surface. The ligand and surrounded residues are shown in sticks. CD1 and HC colors in green, and the surrounded residues are colored in blue.

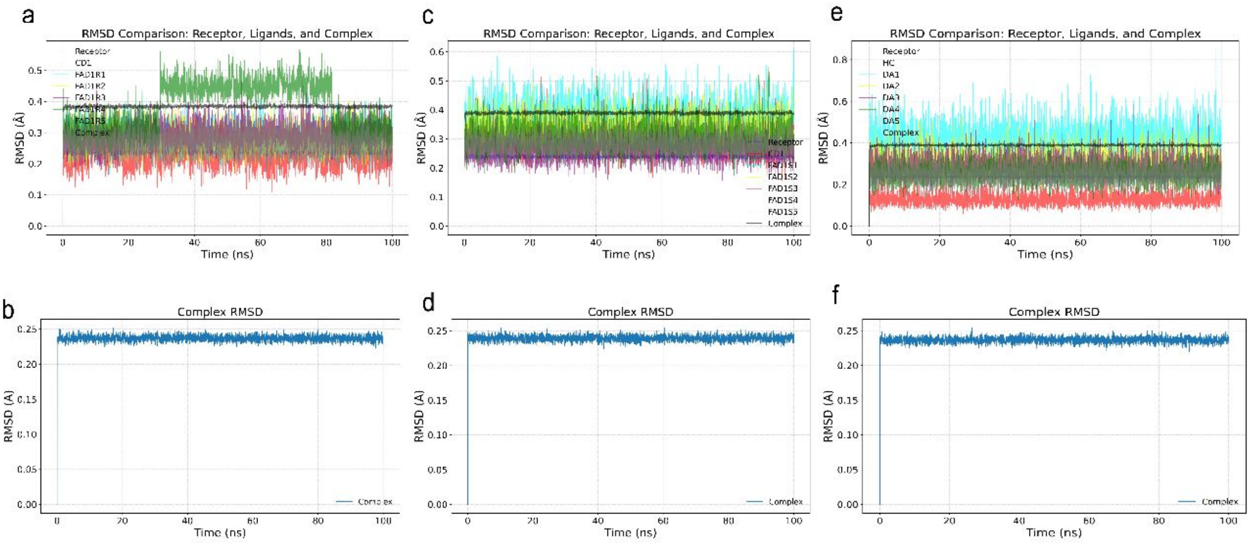

Figure S10. The root mean square deviation (RMSD) of the protein backbone, ligand, and complex of three system. The upper graph represents the RMSD of each individual components of protein backbone, ligand, and complex (**a, c, e**), while the lower graph shows the RMSD of the complex (**b, d, f**). From left to right represent the system of CD1-FAD1 (R) (**a, b**), CD1-FAD1 (S) (**c, d**), HC-DA (**e, f**)

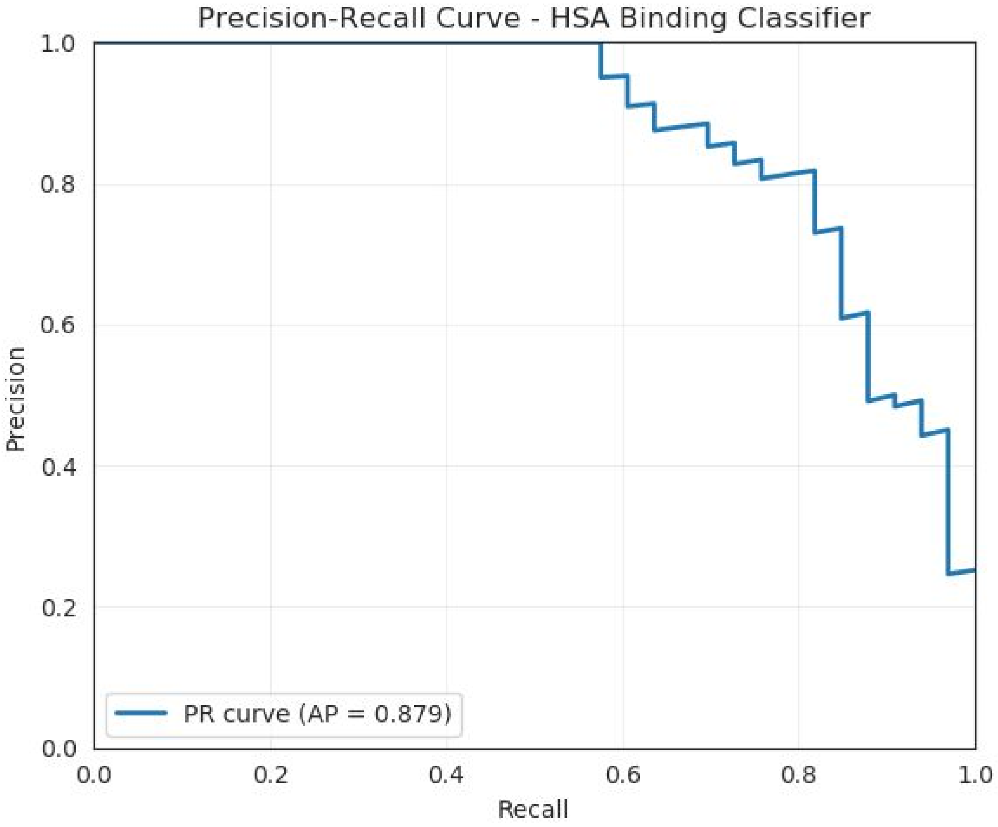

Figure S11. The Precision-Recall Curve of the HSA binding classifier.

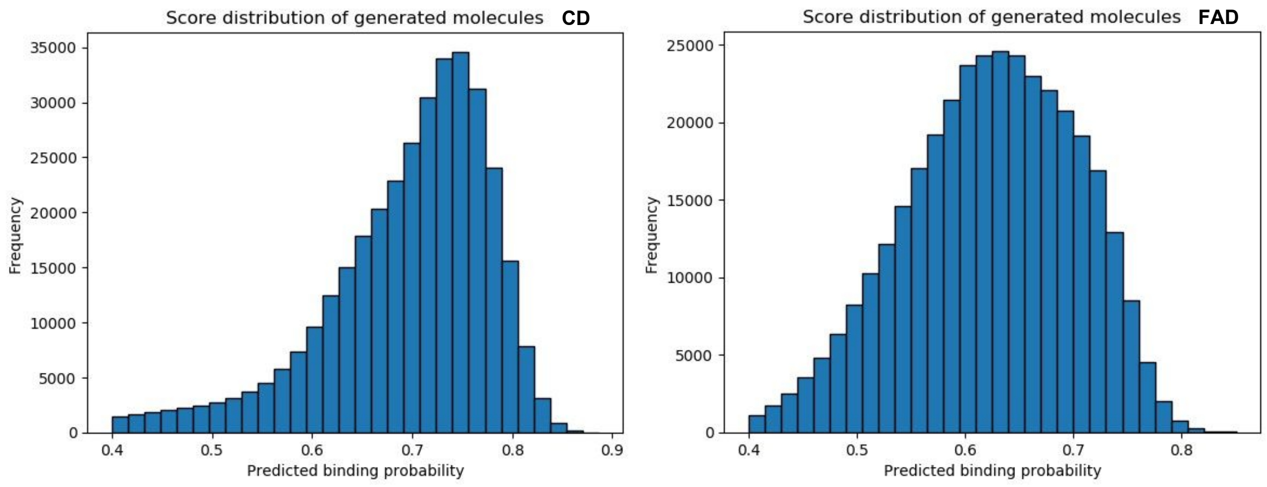

Figure S12. The score distribution of the generated CD and FAD library.

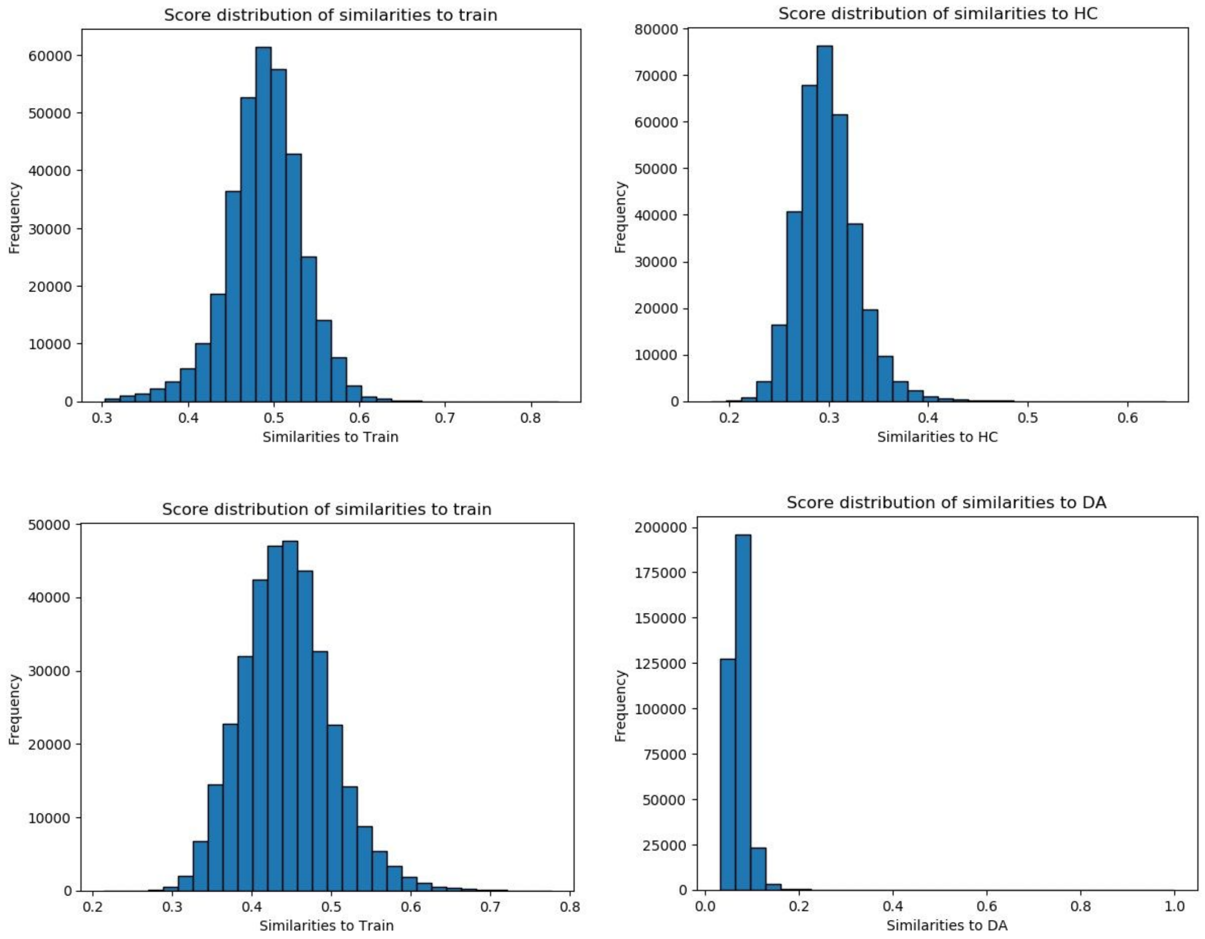

Figure S13. The score distribution of similarities of the generated CD and FAD libraries to train set and to scaffold molecule, respectively.

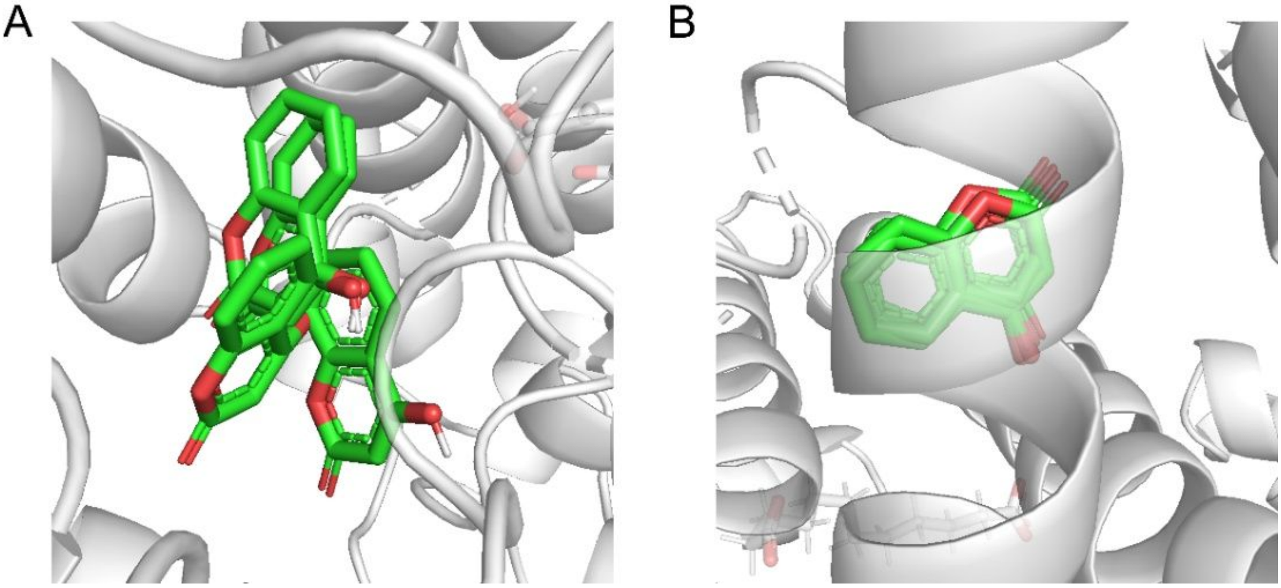

Figure S14. Effect of positional restraints on ligand stability during 100 ns MD simulations.
(A) Superposition of representative structures at 0, 25, 50, and 100 ns from the unrestrained simulationt.
(B) Superposition of representative structures at 0, 25, 50, and 100 ns from the restrained simulation (2 kcal·mol^{-1}·Å^{-2}).

Table S1 The Synthetic Accessibility Score (SAScore) of centroid CD and FAD.

SMILES of FAD	SAScore
O=C(O)CCCCCCCCCC(C(=O)O)c1ccc(-c2ccc(O)c(C(=O)O)c2)c(O)c1	2.89
O=C(O)CCCCCCC(CCCC(=O)O)c1cc(-c2cccc(C(=O)O)c2)c(C(=O)O)cc1O	3.02
O=C(O)CCCCCCC(CCCC(=O)O)c1ccc(Cc2ccc(O)c(C(=O)O)c2)cc1	2.81
O=C(O)CCCCCCCCCCC(=O)c1ccc(O)c(C(=O)O)c1	2.04
O=C(O)CCCCCCCCCCC(=O)c1cc(-c2cccc(C(=O)O)c2)ccc1F	2.15
O=C(O)CCCCCC(CCCCC(=O)O)c1ccc(F)c(F)c1-c1ccc(O)c(C(=O)O)c1	3.08
O=C(O)CCCCCCCC(CCC(=O)O)C(Cc1ccc(O)cc1)c1ccc(O)c(C(=O)O)c1	3.29
O=C(O)CCCCCCCCC(CC(=O)O)c1ccc(O)c(C(=O)c2ccc(C(=O)O)c(O)c2)c1	2.97
O=C(O)CCCCCC(CCCCC(=O)O)c1ccc(-c2ccc(Cl)cc2C(=O)O)c(O)c1	2.90
**SMILES OF CD**	**SAScore**
O=C(O)c1cccc(-c2cccc(-c3cc(=O)oc4ccccc34)c2)c1	1.94
O=C(O)c1cccc(-c2ccc(O)c(-c3cccc4oc(=O)ccc34)c2)c1	2.14
O=C(O)c1cccc(-c2cccc(-c3ccc(-c4ccc5oc(=O)ccc5c4)c(O)c3O)c2)c1	2.40
O=C(O)c1cccc(-c2ccc(O)c(-c3cccc(-c4cc5ccccc5oc4=O)c3)c2)c1	2.23
O=C(O)c1cccc(-c2cc(-c3cc(-c4ccc5ccc(=O)oc5c4)ccc3O)ccc2O)c1	2.42
O=C(O)c1cccc(-c2ccc(-c3ccc(O)c(C(=O)O)c3)c(-c3cccc4ccc(=O)oc34)c2)c1	2.46
O=C(O)c1cccc(-c2ccc(-c3cc(Cl)cc(-c4cccc5oc(=O)ccc45)c3)cc2Cl)c1	2.34
O=C(O)c1ccc(-c2cccc(-c3cccc4oc(=O)ccc34)c2)c(-c2ccc(O)c(C(=O)O)c2)c1	2.40
O=C(O)c1cccc(-c2ccc(F)c(O)c2-c2ccc(O)c(-c3cc(=O)oc4ccccc34)c2)c1	2.54

Table S2. The computational binding free energy for HC, CD1, DA and FAD1 on the binding pocket of HSA.

Energy (kcal/mol)	VDWAALS	EEL	EGB	ESURF	MM/GBSA	Average
HC	-15.1286	89.6738	-92.8416	-2.7864	-21.0828	-21.0828
DA1	-33.2162	85.5015	-75.5418	-5.4397	-28.6963	
DA2	-21.832	228.3944	-232.341	-4.9312	-30.7096	
DA3	-28.0972	3.4322	-45.3388	-5.7583	-75.7621	-53.4703
DA4	-30.3593	20.2914	-31.9108	-5.9217	-47.9003	
DA5	-28.0714	-17.6691	-7.73	-6.0388	-59.5094	
CD1(FAD1R)	-34.0136	161.4801	-160.998	-5.4976	-39.029	-39.029
FAD1R1	-38.7857	213.3474	-199.835	-6.2549	-31.5286	
FAD1R2	-56.0806	478.0658	-461.08	-8.4852	-47.5797	
FAD1R3	-55.15	280.9957	-303.741	-9.4169	-87.3117	-56.7522
FAD1R4	-54.956	338.0613	-316.862	-8.4585	-42.2152	
FAD1R5	-46.1663	114.176	-134.106	-9.0296	-75.126	
CD1(FAD1S)	-30.6096	158.2773	-159.602	-5.2478	-37.1818	-37.1818
FAD1S1	-46.3653	159.9543	-140.682	-7.5334	-34.6259	
FAD1S2	-47.9931	449.1061	-426.036	-8.5695	-33.4924	
FAD1S3	-59.5194	229.7692	-249.592	-9.6275	-88.9696	-58.7015
FAD1S4	-44.9169	243.8364	-256.553	-8.2342	-65.8673	
FAD1S5	-42.5883	119.2614	-138.358	-8.8675	-70.5522	

Table S3. Statistics of the property of the generated CD and FAD library.

Property	CD Library	FAD Library
Number of generated molecules	345,046	350,518
Validity (%)	100	100
Uniqueness (%)	100	100
Novelty (%) (Tanimoto < 0.6)	99.5	99.2
Mean predicted score (mean ± std)	0.696 ± 0.082	0.624 ± 0.079
Avg similarity to nearest training molecule	0.49	0.443
Avg similarity to parent scaffold	0.299 (HC)	0.072 (DA)

Table S4. Statistics of the property of the generated CD1, FAD1, and their scaffold molecules (HC and DA), respectively.

Compound	MW (Da)	cLogP	SPR KD (nM)	MM-GBSA ΔG (kcal/mol)	SAScore
**HC**	162.14	1.5	6.54	-21.1	1.96
**DA**	230.3	3.06	40.52	-53.5	1.56
**CD1**	342.35	4.83	5.02	-38.1	1.94
**FAD1**	458.51	5.23	33.2	-57.7	2.89
